# Contemporary Comprehensive Review on Arsenic-Induced Male Reproductive Toxicity and Mechanisms of Phytonutrient Intervention

**DOI:** 10.3390/toxics10120744

**Published:** 2022-11-30

**Authors:** Mahesh Rachamalla, Joshi Chinthada, Sapana Kushwaha, Sravan Kumar Putnala, Chittaranjan Sahu, Gopabandhu Jena, Som Niyogi

**Affiliations:** 1 Department of Biology, University of Saskatchewan, Saskatoon, SK S7N 5E2, Canada; 2 Facility for Risk Assessment and Intervention Studies, Department of Pharmacology and Toxicology, National Institute of Pharmaceutical Education and Research, S.A.S Nagar 160062, India; 3 Department of Pharmacology and Toxicology, Transit Campus, National Institute of Pharmaceutical Education and Research-Raebareli, Lucknow 226002, India; 4 Toxicology Centre, University of Saskatchewan, Saskatoon, SK S7N 5E2, Canada

**Keywords:** arsenic, metalloid, phytonutrients, reproductive toxicity, molecular mechanisms, reproductive failure

## Abstract

Arsenic (As) is a poisonous metalloid that is toxic to both humans and animals. Drinking water contamination has been linked to the development of cancer (skin, lung, urinary bladder, and liver), as well as other disorders such as diabetes and cardiovascular, gastrointestinal, neurological, and developmental damage. According to epidemiological studies, As contributes to male infertility, sexual dysfunction, poor sperm quality, and developmental consequences such as low birth weight, spontaneous abortion, and small for gestational age (SGA). Arsenic exposure negatively affected male reproductive systems by lowering testicular and accessory organ weights, and sperm counts, increasing sperm abnormalities and causing apoptotic cell death in Leydig and Sertoli cells, which resulted in decreased testosterone synthesis. Furthermore, during male reproductive toxicity, several molecular signalling pathways, such as apoptosis, inflammation, and autophagy are involved. Phytonutrient intervention in arsenic-induced male reproductive toxicity in various species has received a lot of attention over the years. The current review provides an in-depth summary of the available literature on arsenic-induced male toxicity, as well as therapeutic approaches and future directions.

## 1. Introduction

Arsenic (As) is a naturally occurring toxic metalloid with odourless, colourless, and tasteless properties. The most commonly available forms of arsenic include inorganic As, organic As, and arsine gas [1]. Arsenic is ranked as the 20th most abundant element in the terrestrial, 14th in the marine, and 12th in the human ecosystem. It possesses significant health concerns due to its ubiquitous existence, discharged into the environment from volcanic and industrial activities [2,3]. Charles Dickens used arsenic as a tonic, called Fowler’s solution (potassium arsenate in water). It was also employed to treat leukaemia, psoriasis, and chronic bronchial asthma [4,5]. As is a well-known poison whose use is restricted to the production of pesticides, herbicides, cotton desiccants, and exfoliants in agriculture, applied as doping material in the semiconductor industry, bronzing, pyrotechnics, as well as the manufacturing of special kinds of glasses and preservation of wood [6,7]. As is found naturally in both trivalent and pentavalent forms, but the pentavalent form is much more common and is the best form for eliminating arsenic from biological systems [8,9]. The commonly available pentavalent forms of arsenic are arsenic pentoxide, arsenic acid, sodium arsenate, lead arsenate, and calcium arsenate [10,11]. More than 200 million people are adversely affected by toxic responses to As, which acknowledges research and mitigation methods to control human arsenic exposure [12]. Since it is ubiquitous, humans are exposed via groundwater, food, and industrial and anthropogenic sources [13]. The largest source of As exposure for people is drinking water; the World Health Organization (WHO) and Environmental Protection Agency (EPA) recommend a maximum concentration of 10 µg/L for As in drinking water [14]. As exposure through the air is negligible, effects are observed when the air comprises a mixture of arsenite and arsenate [14]. As mobilisation in drinking water results from microbial reactions such as oxidizing arsenite or reducing arsenate [15]. Furthermore, arsine gas, gallium arsenide, glass manufacturing facilities, and coal-fired power plants are some of the occupational sources of arsenic [16]. Furthermore, elevated levels of arsenic were found in commonly consumed foods such as grains, vegetables, and rice, as well as significant concentrations in meat products such as beef, poultry, and shellfish [17]. Several countries around the world are highly exposed and vulnerable to arsenic toxicity, including Cambodia, China, India, Mexico, Pakistan, the United States, Vietnam, and East Croatia [18,19,20]. Chronic exposure in Bangladesh and Taiwan, as well as low-level exposure in the United States, has resulted in the onset of type II diabetes [21,22]. Pregnant women in the Chilean region who were exposed to As in their drinking water for an extended period of time experienced a decrease in baby birth weight [23]. Cancer, genetic changes, and dermatological diseases have been reported in Argentina when As concentrations reach 100 µg/L [24]. In Taiwan, As contamination in drinking water increases the risk of lung, kidney, and bladder cancer [25]. As levels in the aquifer have the greatest impact on a few provinces in India (Uttar Pradesh, Bihar, and West Bengal) and Bangladesh [26,27,28,29].

As is designated by the International Agency for Research on Cancer (IARC) as a group-I carcinogen that also raises the risk of bladder, lung, kidney, and liver cancer [25,30,31,32].

As induces carcinogenesis by epigenetic modifications in miRNA expression [33,34], DNA methylation, and histone modifications [35]. Low doses of As in the form of orpiment (AS_2_S_3_), realgar (AS_4_S_4_), and especially arsenolite (contains arsenic trioxide, AS_2_O_3_) have been used as a therapeutic agent in Iranian traditional medicine since Avicenna (1023 A.D.) [36,37]. Interestingly, As was therapeutically used to treat chronic myelogenous leukaemia (CML) until radiation and chemotherapy took over [9]. As has also demonstrated significant success in the treatment of newly diagnosed and relapsed individuals with acute promyelocytic leukaemia (APL) [38]. Acute exposure to As causes nausea, vomiting, abdominal pain, and severe diarrhoea, whereas prolonged exposure causes damage to multiple organs [11,39]. Interestingly, Calabrese and Baldwin (2009) found hormetic dose–response relationships with inorganic compounds including arsenic showed that mechanisms associated with arsenic may decipher the new dimensions. Recently, Ommati et al., found that in mature F1 male mice, the spermatogenic index was higher in low-dose (0.2 ppm) animals, demonstrating another possible hormesis effect, but a dose-dependent decrease in the spermatogenic index was found at higher doses (2 and 20 ppm) of As_2_O_3._ It is interesting to note that Calabrese and Baldwin (2009) discovered hormetic dose–response relationships with inorganic compounds, one of which was arsenic. These relationships revealed that mechanisms associated with arsenic may be able to decipher new dimensions [40]. Ommati et al. (2019) recently found that in mature F1 male mice, the spermatogenic index was higher in low-dose (0.2 ppm) animals, demonstrating another possible hormesis effect, whereas a dose-dependent decrease in the spermatogenic index was found at higher doses (2 and 20 ppm) of As_2_O_3_ [41]. In addition, a hormesis effect was seen in female mice, as indicated by a decrement in thiobarbituric acid reactive substances (TBARS) content and an increase in ovarian mammalian target of rapamycin (mTOR) gene expression level, both of which occurred at lower doses of As_2_O_3_ [42]. They also observed the hormesis effect in the hypothalamic–pituitary–gonadal (HPG axis) of pubertal male offspring when exposed to low levels of As_2_O_3_ (0.2 ppm) [37].

The parental mice in this study were exposed to As_2_O_3_ (0, 0.2, 2, and 20 ppm) starting five weeks before mating and continuing until the male pups reached puberty. After the exposure period, the HPG axis experienced increased oxidative stress and autophagy, particularly at higher As_2_O_3_ doses (2 and 20 ppm). The number of MDC-labelled autophagic vacuoles and MDA/GSH ratio in the HPG axis of pubertal F1 male mice exposed to higher As_2_O_3_ doses increased, whereas mean body weight, total antioxidant capacity, and stereology indices decreased. Meanwhile, in pubertal F1 male HPG tissues, in addition to a dose-dependent increase in ATG3, ATG5, Beclin gene expression, and protein expression of P62, ATG12, and Beclin, a dose-dependent decrease in PI3K and mTOR gene expression was observed. Higher doses of As_2_O_3_ appear to impair HPG axis functionality in pubertal male mice offspring by increasing the MDA/GSH ratio, autophagic cell death-related genes and proteins, and decreasing total antioxidant capacity.

In addition, chronic As exposure causes hyperpigmentation in humans [43,44]. Several lines of evidence point to As having negative consequences such as impaired cognitive function [11], developmental neurotoxicity [45], hemopoietic and immune system suppression [46,47], skeletal muscle damage [48], the onset of diabetes [49] reproductive dysfunctions [50], and spleen damage [51]. As was found to accumulate in several organs like the eye [52], kidney, and liver [53] and it was also shown in brown adipose tissue, where it was demonstrated to inhibit adipogenesis, mitochondrial biogenesis, and thermogenesis [54]. Perinatal exposure to As showed depressive behaviours in adult offspring [55] and was prone to neurodegenerative disorders [56]. As is an endocrine-disrupting agent [57] and a well-known reproductive toxicant that induces neural tube defects in laboratory animals [58]. Because of its higher distribution to reproductive organs, it has been shown to have a high incidence of reproductive toxicity, particularly testicular toxicity in males. Subchronic exposure has been linked to poor sperm quality and spermatogenesis [17,40,41,42] and genotoxicity in testicular cells [59]. Sperm motility, morphology, and viability were all affected when mice were exposed to sodium arsenite acid phosphatase, alkaline phosphatase, and lactate dehydrogenase testicular activity, steroidogenic genes, such as the steroidogenic acute regulatory (StAR) protein and the cytochrome P450 side-chain cleaving enzyme (P450scc; Cyp11a) 3β-Hydroxysteroid dehydrogenase (3β-HSD) and 17β-Hydroxysteroid dehydrogenase (17β-HSD) were also affected [60]. Male mice exposed to arsenic for 3 and 6 months develop a compromised gut microbiome and an increase in inflammatory cytokines and immune cells and increased colon cancer markers β-catenin [61]. Moreover, recent studies also suggest that arsenic altered the gut microbiota [62,63]. So, if there is a change in gut microbiota, as suggested by these findings, it may be associated with testicular dysfunction [64,65].

In the last few years, there has been significant improvement in phytonutrient intervention [66,67] in various toxicities which proved to be promising in ameliorating arsenic and other pollutants toxicity [68,69,70]. Because the majority of arsenic’s negative effects are caused by the development of oxidative stress, antioxidant treatment has proven to be a successful method of combating its toxic effects. Because arsenic affects the intracellular antioxidant system, phytonutrients, also known as exogenous antioxidant supplements, may counteract the pro-oxidant stress caused by arsenic. The antioxidant properties of several flavonoids have been shown to be beneficial in reducing the harmful effects of arsenic. The goal of this paper is to combine data from animal and human studies, as well as to review and summarise the molecular mechanisms of As-induced male reproductive toxicity. Furthermore, this review focuses on different phytonutrients and their mechanisms in reducing As-induced male reproductive toxicity.

### 1.1. Exposure to Arsenic in the Environment

Arsenic exposure in humans is constantly rising in drinking water, food, and industrial sources. Higher arsenic levels were reported in drinking water in most countries and were higher than the specified limits of WHO and EPA. Cultivation of rice with As-contaminated soil and the use of As-contaminated water to cook rice are the major contributors to As exposure in cooked rice [71]. Finfish, shellfish, and seaweed are the most common sources of As exposure in humans through seafood [72]. As depicted in Table 1, urine, hair, and drinking water samples collected from children and adults from As-contaminated regions in Argentina, Uruguay, India, Pakistan, and Spain showed higher levels of arsenic. Accumulation levels of arsenic in various foods like rice, meat, cereals, and vegetables in different regions of the world were presented in Table 2. Overall, there is a significant risk of cancer in children and adults due to the build-up of arsenic in the diet.

### 1.2. Metabolism of Arsenic

**Figure 1:**
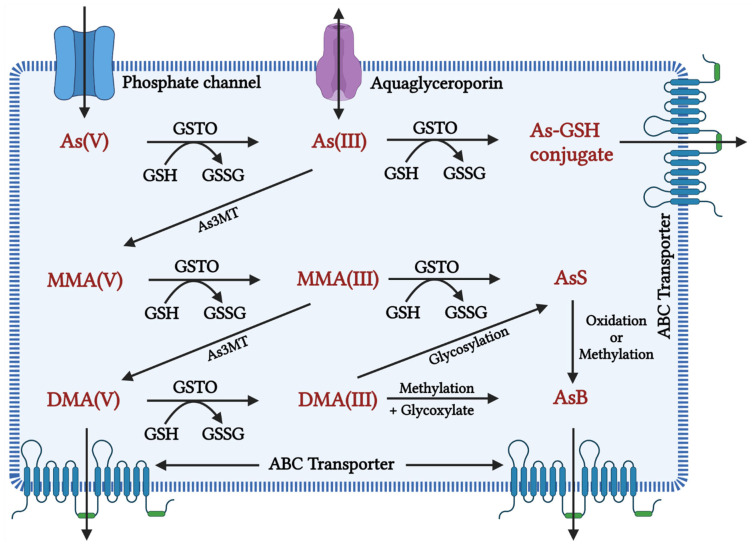
Inorganic arsenic conversion pathways into mono-, di-, and trimethylated products when the toxic form of As(III) is taken up by a cell, it is methylated to MMA (monomethylarsonic acid) and DMA (dimethylarsinic acid) with the help of the enzyme arsenic methyl transferase (AsMT) and can then be eliminated from the body through urine. However, in a pathological state, SAM depletion causes As(III) aggregation, affecting cellular homeostasis in a variety of ways, including oxidative balance, inflammation, and genetic and epigenetic processes. This disruption in homeostasis causes cellular damage and, eventually, cell death. As(V): inorganic pentavalent arsenic; As(III): inorganic trivalent arsenic; As3MT: arsenite methyltransferase, MMA(V): methyl arsonate; MMA(III): monomethylarsonous acid; DMA(V): dimethyl arsenate; DMA(III): dimethylarsinous acid; GSH: glutathione; GSTO: Glutathione S-transferase omega, GSSG: Glutathione disulfide As-GSH: Arsenic glutathione; AsS: Arsenic sulphide; AsB: Arsenobetaine; ABC transporter: ATP-binding cassette transporters; AS3MT: arsenite methyltransferase.

In many organisms, along with humans, the inorganic form of As gets rapidly reduced from arsenate (pentavalent) to arsenite (trivalent) in the blood, facilitated by glutathione (GSH) [86]. The latter is 2–10 folds more toxic and rapidly taken up by the cells than the former [10]. Once absorbed, arsenite binds to globin of Hb- and -SH-containing proteins such as Glutathione and Cysteine and is distributed to the skin, hair, and mucosa owing to thiol-cystine amino acids. Skin, hair, epithelium of gastrointestinal tract (GIT), and epididymis had the highest As retention [87,88,89,90]. Aquaglyceroporins (AQPs) are involved in transporting As into cells [91], whereas efflux is carried out by major facilitator superfamily (MFS) transporters and ATP-binding cassette (ABC) transporters [92]. As shown in Figure 1, initially, biotransformation of arsenic involves oxidative methylation of arsenite to arsenate, mediated by the enzyme arsenite methyltransferase (As3MT), a primary methyl donor, followed by oxidation, arsenite to a pentavalent metabolite of arsenate, monomethylarsonic acid (MMA), and dimethylarsinic acid (DMA) [93,94]. MMA and DMA were taken up less effectively by organs and tissue than arsenite and rapidly excreted into urine [95]. In contrast, evidence suggests that metabolites of As tend to induce chromosomal mutations [96] and show genotoxicity [97]. Methylated forms of As were also found in human urine, natural water, and bird eggshells [98].

### 1.3. Arsenic-Induced Oxidative Stress and DNA Damage

**Figure 2:**
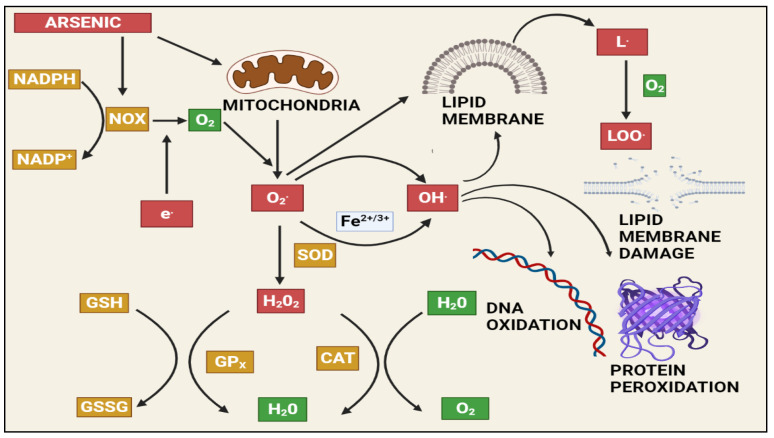
The effects of arsenic-induced mitochondrial reactive oxygen species generation. Arsenic generates a significant amount of ROS, primarily through complexes I and II of the electron transport chain (ETC). The ETC produces superoxide radicals, which react with other radicals in the cell to form stable and long-lived reactive species that damage macromolecules and induce apoptosis via various pathways. NAPDH: Nicotinamide adenine dinucleotide phosphate (NADP+), NADPH oxidase (NOX), GSH: Gluthathione, GSSG: Glutathione disulfide, GPx: Glutathione peroxidase, SOD: Superoxide dismutase, CAT: Catalase, H_2_O_2_: Hydrogen peroxide, LOO: lipid peroxy radical.

At a biochemical level, inorganic arsenic in the pentavalent state replaces the phosphate in several reactions, the trivalent form of inorganic and organic (methylated) arsenic reacts with critical thiols and inhibits their activity [93]. As induces the formation of reactive oxygen species (ROS), enhances free radicals, and diminishes the activity of thiol group-rich antioxidants such as *glutathione* (GSH) [99]. Production of ROS will take place during the generation of intermediate arsine species [100]. Methylated arsenicals cause the release of iron from ferritin and initiate the production of hydroxy radicals and progress ferroptosis [101]. Arsenic shows mitochondrial toxicity by inhibiting succinic dehydrogenase activity and uncoupling oxidative phosphorylation with the production of O_2,_ which gives rise to other forms of ROS [102]. Oxidative stress leading to the activation of ERK/AKT/NF-kβ pathway is one of the primary molecular mechanisms involved in arsenic-induced male reproductive toxicity [10]. Mitogen-activated protein kinases (MAPKs) are proteins that regulate the signalling of cells in response to environmental stimuli. The ERK signalling pathway is involved in a variety of male reproductive functions, such as spermatogenesis and Sertoli cell function. Activation of ERK1/2 inhibits the function of Sertoli cells and increases apoptosis in the testes. Protein kinase B (AKT) regulates oxidative stress in conjunction with the immune system by regulating cell growth, survival, proliferation, and inflammation. MAPK and AKT both directly phosphorylate nuclear factor kappa B (NF-B), which increases NF-ĸB binding to target DNAs and the expression/activity of NF-ĸB-controlled genes. Spermatogenesis and the function of Sertoli cells in the testes are governed by the transcription factor NF-ĸB. Activation of NF-ĸB inhibits spermatogenesis in both humans and mice. Arsenic activated the ERK/AKT/NF-B signalling pathways in numerous cell types. The expression of ERK1/2, IKK, PI3K, AKT, and NF-B, as well as the phosphorylation of ERK/AKT, increased in rats exposed to sodium arsenite (1, 5, or 25 mg/L) for 6 months. This causes reproductive toxicity via ERK/AKT/NF-kB signalling [68,69,70,103,104,105,106,107]. Figure 2 depicts As-induced oxidative stress mechanisms.

### 1.4. Effects of Arsenic on the Male Reproductive System in Animals

**Figure 3:**
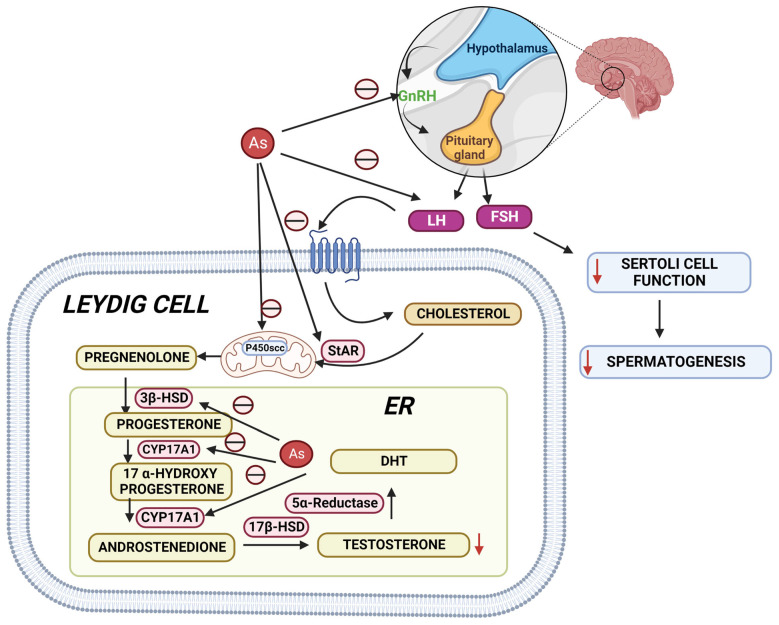
Arsenic’s mechanisms show that it alters the hypothalamic–pituitary–gonadal (HPG) axis, resulting in a decrease in testosterone biosynthesis, Sertoli cell activity, and spermatogenesis. StAR: steroidogenic acute regulatory protein; 3HSD: 3-beta (β)-hydroxysteroid dehydrogenase; CYP17A1: Cytochrome P450 17A1; 17HSD: 17-beta (β)-hydroxysteroid dehydrogenase; DHT: Dihydrotestosterone.

Epidemiological studies have suggested that arsenic is one of the most hazardous reproductive toxicants present in the environment, which is significantly accumulated in the reproductive tissues like the testes, epididymis, seminal vesicle, and prostate gland [108,109]. The inhibition of testosterone biosynthesis by arsenic is depicted in Figure 3. Arsenic exposure exerted cellular and molecular perturbations such as oxidative stress, inflammation, induction of autophagy, and apoptosis, which obstructed male gonadal development and led to reproductive dysfunction in humans and animals [110,111]. Table 3 Shows studies on arsenic-induced reproductive toxicity in animals. Arsenic has been shown to damage the histology of various tissues, such as the liver, brain, and kidney [112,113]. Notably, arsenic exposure during development significantly altered tight junctions’ proteins, leading to an increase in blood–brain barrier permeability [114]. As a result, the age-dependent inhibition of the PI3K/Akt/mTOR signalling pathway may contribute to the induction of autophagy and the facilitation of arsenic transfer through the cerebellum’s cerebral cortex and hippocampus leaky blood–brain barrier [115]. Recent studies from the Ommati research group also shed some light on hypothalamic–pituitary–gonadal (HPG) axis disruption and subsequent toxicity in mice and its continued impact on offspring as well (refer to Figure 3) [37,41,43,116,117]. Similarly, the blood–testis barrier (BTB) is one of the most impermeable blood–tissue barriers in mammals. It divides the seminiferous epithelium into basal (intraluminal) and apical (intraluminal) compartments. Meiosis I and II, spermiogenesis, and spermiation all occur in a specialised microenvironment behind the BTB in the apical compartment, whereas spermatogonial renewal and differentiation, as well as cell cycle progression up to the preleptotene spermatocyte stage, occurs outside the BTB in the basal compartment of the epithelium. However, the BTB is not a static ultrastructure. Instead, it undergoes extensive remodelling during stage VIII of the seminiferous epithelial cycle of spermatogenesis to allow preleptotene spermatocytes to pass through the BTB. However, the BTB’s immunological barrier cannot be compromised, even temporarily, during the epithelial cycle in order to prevent the production of antibodies against meiotic and postmeiotic germ cells. Adhesion protein complexes (e.g., occludin-ZO-1, N-cadherin—catenin, claudin-5-ZO-1), steroids (e.g., testosterone, estradiol-17), nonreceptor protein kinases (e.g., focal adhesion kinase, c-Src, c-Yes), polarity proteins induce testicular damage via their initial actions at the BTB, resulting in germ-cell loss, reduced sperm count, and male infertility or subfertility. Metallothioneins [cysteine-rich low molecular weight metal-binding proteins localised to the membrane of the Golgi apparatus that protect cells from cytotoxicity of essential heavy metals (such as zinc, selenium, and copper) and non-essential heavy metals (such as arsenic, mercury, silver, and cadmium) by binding to these metals via the thiol groups] are largely responsible for heavy metal accumulation in the body. As a result, significant and harmful amounts of heavy metal accumulation can accumulate in a person over time, exceeding the capacity of metallothioneins in the process [118,119,120,121,122,123].

Testicular histopathological investigations showed that sodium arsenite decreased seminiferous tubule diameter in Wistar rats [16] and outbred Institute of Cancer Research (ICR) mice led to a significant decrease in the lumen in the arsenic-treated group compared to the control group [124]. Vacuolisation, acidophilic cells, and epithelial degeneration were associated with increased inflammatory cytokines in male rats exposed to sodium arsenite through drinking water [125]. Moreover, sodium arsenite exhibits severe damage to the testicular structure and elevated cleaved caspase 3 (CC3) which is an apoptotic marker in the cluster differentiation 1 (CD1) mouse testes cell line [126]. Increased expression of CC3 also indicates increased apoptosis. Together, sodium arsenite and arsenate at a concentration of 0.01 and 10 mg/L in drinking water showed a decrease in catalase activity and vacuolisation of seminiferous tubules in rats [127]. Arsenic exposure inhibited the spermatogenesis process and decreased the mobility and viability of sperm [128]. An increase in apoptotic spermatozoa has been observed with arsenic exposure, the central mechanism involved in arsenic-induced decreased sperm count [129]. Sodium arsenite exposure at a concentration of 10 mg/L through drinking water for eight weeks exhibited a decrease in sperm counts and enhanced sperm head abnormalities, which led to an increase in the infertility risk and pre-implantation loss in Wistar rats [130]. Moreover, in mice, arsenic trioxide at doses of 0.3 and 3 mg/kg s.c. for 35 days reduced the number of spermatozoa, increased epithelial aberration and exfoliation of germ cells in the tubule lumen, and altered the nucleus/cytoplasm ratio of Leydig cells [131]. In mouse testes, arsenic trioxide at concentrations of 0.2, 2, and 20 ppm in drinking water for six months impaired sperm motility and affected the ultra-structure of the acrosome structure and sperm tail by downregulating the protein expression of DPY19L2, AKAP3, CFAP44, and SPAG16 [132]. Similarly, arsenic trioxide inhibited the expression of the DDX25 and CRM1 mRNAs, as well as the downstream proteins HMG2, PGK2, and H4 necessary for spermatogenesis in mice [133]. Oral exposure to arsenic trioxide at doses of 0.3 and 30 µg/kg for 15 days resulted in a dose-dependent frequency of sperm production in mice with aberrant head morphology [134]. Diabetic rats exposed to sodium arsenite at a concentration of 10 mg/L in drinking water for 40 days caused a drop in serum testosterone, sperm counts, motility, morphology, and acrosomal and plasma membrane structure [135]. Decreases in sperm count and viability with increased arsenic accumulation, lipid peroxidation, and protein carbonylation in the testes have been observed by administration of sodium arsenate at a concentration of 10, 25, 50, 100, and 200 ppm for 40 days through drinking water in mice [136]. Rats exposed to sodium arsenite at doses of 1, 5, and 25 mg/L through drinking water for 6 months showed compromised sperm counts and motility, and testosterone and altered 19 proteins related to reproduction such as Vdac3, Prkaca, Hspa41, Spaca1, Ma1b, Gpx4, Safb1, Trim28, Rbp1, Hsd11b1, Mapk3, Gpd2, Ace, Hspa11, Dnaja1, Ybx3, Smcp, Nasp, and Cabs1 were altered [104].

### 1.5. Pre-Natal Arsenic Exposure Induced Male Reproductive Toxicity in Animals

The growing evidence suggests that parental and/or pre-natal arsenic exposure to animals resulted in post-natal developmental toxicity. In-utero exposure to sodium arsenite from embryonic day-10 to day-18 at a concentration of 10 ppb induced an increase in leptin levels, and at 42.5 ppm reduced the litter size compared to control mice [137]. Chronic As trioxide exposure to parental male at a dose of 1 mg/L showed genotoxic damage in F0-F3, altered methylation patterns, changes in reproductive parameters, morphological damage in the ovaries (F0 and F1) and testicles (F1–F3), and compromised sperm quality (F0-F3, except F2) [138]. Exposure of sodium arsenite at the dose of 10 mg/L in drinking water to pregnant females from GD1-21 affected body weight and initial sexual development in male pups and relative anogenital distance also showed changes in the expression of SOD1, SOD2, CAT, and GSTK1 gene in male pup rat [139]. Administration of sodium arsenite at a concentration of 10 mg/L showed lower sperm production, sperm count, motility, and quality in the epididymis of rats [140]. Daily sperm production and the number of spermatids in the rat epididymis were reduced after oral treatment of sodium arsenite at doses of 0.01 and 10 mg/L for 56 days [141]. Lead at the dose of 819 mg/L was exposed to pregnant rats through drinking water until weaning, followed by the exposure of arsenic to male offspring at a dose of 2.3 mg/L, which showed a decrease in daily sperm production, relative weights of testes, epididymis, seminiferous tubules, and prostate, and decreased the activity of 3β-HSD and 17β-HSD in male offspring [142]. Sodium fluoride and sodium arsenite at 100 mg/L and 50 mg/L, respectively, via drinking water during the pre-pregnancy period, decreased the testicular weights, serum FSH, LH, and testosterone levels, and increased Beclin1 and LC3 expressions in Sprague Dawley (SD) rats’ testes [143].

**Table 3:** Preclinical and cell line studies show that arsenic causes reproductive toxicity and alters testicular functions.

S. No	Test Organism	Arsenic Species	Exposure Regimen	Route of Exposure	Duration of Exposure	Organ/Tissue/Cell Line	Observations	Reference
RODENTS								
**1**	Mice	As trioxide	0.3 and 3 mg/kg bw	Subcutaneous	35 days	Testes	Decreased sperm count, increased seminiferous tubule’s epithelial aberration, and exfoliation of germ cells, altered nucleus/cytoplasm ratio of Leydig cells.	[131]
**2**	Mice	As trioxide	0.2, 2, and 20 mg/kg bw	Drinking water	180 days	Testes	Reduced sperm motility, altered ultra-structure of acrosome and sperm tail.	[132]
**3**	Mice	As trioxide	0.2, 2, and 20 mg/kg bw	Oral	180 days	Testes	Reduced spermatid elongation, decreased DDX25 and CRM1 mRNA expression and HMG2 and PGK2 proteins expression.	[133]
**4**	Mice	As trioxide	0.0003, 0.0015, 0.015, and 0.03 mg/kg bw	Oral	15 days	Sperm	Abnormal head morphology.	[134]
**5**	Mice	Sodium arsenate dibasic heptahydrate	10, 25, 50, 100, and 200 mg/kg bw	Oral	40 days	Testes and sperm	Decreased sperm kinetics, viability, plasma membrane integrity. Altered SOD, CAT, and GST levels. Reduced sperm count.	[136]
**6**	Mice	As trioxide	0.2, 2, 20 mg/kg bw	Oral	≈123 days	Testes	Enhanced PI3K, Atg5, Atg12 gene expressions, Increased Beclin1, LC3-I, LC3-II, and p62 protein expressions (F1 generation).	[41]
**7**	Mice	As trioxide	0.2, 2, 20 mg/kg bw	Oral	≈123 days		Increased number of MDC-labeled autophagic vacuoles, and MDA/GSH ratio in HPG axis of pubertal F1 male in highest dose treated animals. A dose-dependent increase expression of ATG3, ATG5, Beclin genes, protein expression of P62 ATG12, and Becline. Decreased gene expression of PI3K and mTOR gene expression was recorded in the HPG tissues of puberty F1 males.	[37]
**8**	Mice	Sodium arsenite	0.05 and 1 mg/kg bw	Oral	1, 2, and 3 days	Testes	Cytotoxicity and disrupted antioxidant mechanisms in the Leydig cells and Sertoli cells.	[144]
**9**	Mice	Sodium arsenite	5 and 50 mg/kg bw	Oral	180 days	Testes	Reduced LHR, StAR, 3β-HSD, and 17β-HSD expression. Downregulation of StAR, 17β-HSD, and Ddx3y mRNA.	[145]
**10**	Mice	As trioxide and antimony	4 and 15 mg/kg bw	Oral	60 days	Testes and sperm	Altered sperm count, morphology, survival, testosterone level. Reduced germ cell count, T-AOC, SOD, and MsrB 1 levels. Upregulation of Beclin1, Atg-5, LC3B/LC3A, Caspase-8, Cytc, CC-3, p53, Bax	[146]
**11**	Diabetic rats	Sodium arsenite	10 mg/L	Oral	40 days	Testes and sperm	Decreased serum testosterone, daily sperm production, motility, and morphology. Impairment of acrosome and plasma membrane integrity.	[135]
**12**	Rat	Sodium arsenite	0.01 and 10 mg/L	Oral	32 days (PND 21 to PND 53)	Testes	Increased vacuolisation, acidophilic cells, and epithelial degeneration. Increased testicular fluid and inflammatory infiltration.	[125]
**13**	Rat	Sodium arsenite	5 mg/kg bw	Oral	56 days	Testes	Decreased testicular weights and seminiferous tubule diameter	[16]
**14**	Rat	Sodium arsenite	10 mg/L	Oral	56 days	Testes	Decreased sperm counts and enhanced sperm head abnormalities. Infertility risk and pre-implantation loss.	[130]
**15**	Rat	Sodium arsenite	1, 5 and 25 mg/L	Oral	180 days	Testes	Down-regulation Lhr, Star, P450scc, Hsd3b, Cyp17a1, Hsd17b, and Aromatase mRNA expressions. Upregulation H3K9me3 methyltransferase, Suv39h1. Down-regulation of demethylase and Jmjd2a.	[147]
**16**	Rat	Sodium arsenite	10 mg/L	Oral	30 days (PND 21 to 51)	Testes and epididymis	Overexpression of SOD1, SOD2, CAT, GSTK1, and MT1 in testes and SOD1, CAT, and GSTK1 in epididymis.	[148]
**17**	Rat	Sodium arsenite and arsenate	0.01 and 10 mg/L	Oral	56 days	Testes	Decreased CAT activity. Increased vacuolisation in seminiferous tubule.	[127]
**18**	Rat	Sodium arsenite	0.01 and 10 mg/L	Oral	20 days (PND 23 to 53)	Prostate	Tissue damage and delayed maturation of prostate.	[147]
**19**	Rat	Sodium fluoride and sodium arsenite	100 and 50 mg/L	Oral	113 days	Testes	Reduced FSH, LH, and testosterone levels. Increased Beclin1 and LC3 expression. Decreased p62 expression.	[143]
**20**	Rat	Lead and sodium arsenite	819 and 2.3 mg/L	Oral	60 days (PND 55–115)	Testes	Decreased reproductive organ weights and daily sperm production. Decreased 3β-HSD and 17β-HSD activities.	[142]
**21**	Rat	Sodium arsenite	1, 5, 25 mg/L drinking water	Oral	180 days	Reproductive parameters	Compromised sperm counts and motility, serum testosterone. Alteration in proteins related to reproduction such as Vdac3, Prkaca, Hspa41, Spaca1, Ma1b, Gpx4, Safb1, Trim28, Rbp1, Hsd11b1, Mapk3, Gpd2, Ace, Hspa11, Dnaja1, Ybx3, Smcp, Nasp, Cabs1.	[104]
**22**	Rat	As trioxide	1 mg/mL	Oral	112 days	Testes	Alterations in methylation patterns and reproductive parameters. Morphological aberration in ovaries (F0 and F1) and testicles (F1–F3). Decreased sperm quality (F0–F3, except F2).	[138]
**23**	Rat	Sodium arsenite	10 mg/L	Oral	21 days (GD 1–21)	Testis and Epididymis	Changes in SOD1, SOD2, CAT, and GSTK1 gene expression. Altered SOD, Catalase, and GSH activities.	[139]
**24**	Rat	Sodium arsenite	0.01 and 10 mg/L	Oral	56 days	Epididymis	Reduced daily sperm production, number of spermatids	[127]
**25**	Rat	Sodium arsenite	10 mg/L	Oral	30 days (PND 52 to PND 81)	Epididymis	Lower sperm production, sperm count, motility and quality.	[140]
**CHICKEN**								
**26**	Chicken	As trioxide	7.5, 15, and 30 mg/kg bw	Oral	30, 60, and 90 days	Testes	Increased NF-Kβ, TNF-α, i-NOS, COX-2, and PTGEs mRNA over expressions. Increased Hsp70 and HSp90 mRNA expressions.	[149]
**27**	Chicken	Copper sulphate and As trioxide	300 and 30 mg/kg bw	Oral	28, 56, and 84 days	Testes	Increased mRNA levels of pro-inflammatory cytokines and inflammatory factors. Increased mRNA and protein levels of Hsp60, Hsp70, and Hsp90	[150]
**28**	Chicken	As trioxide	0.625, 1.25, and 2.5 mg/kg bw	Oral	30, 60 and 90 days	Testes	Enhanced LC-III, dynein, Beclin-1, ATG-5, and ATG4B expression.	[151]
**CELL LINE**								
**29**	MLTC-1 Line	As trioxide	3, 6 and 9 µM	NA	1 day	Leydig cell	Accumulation of autophagosomes.	[152]
**30**	MLTC-1 Line	Sodium arsenite	1, 2, and 4 mg/L	NA	2 days	Leydig cell	Enhanced mRNA and protein expression levels of 3β-HSD by suppressing H3K9me2/3, whereas genes Star, P450scc, P45-c17, and 17β-HSD were downregulated.	[153]
**31**	GC-1 Spermatogonial (SPG) cell line	As trioxide	10, 20 µM	NA	1 day	Spermatogonia	Damaged mitochondria upregulated ATG3, p62, LC-3I, and LC-3II mRNA expressions.	[154]

## 2. Mechanisms of Arsenic-Induced Male Reproductive Toxicity in Animals

The major toxicity mechanisms include inflammatory response, oxidative stress, autophagy, and apoptosis.

### 2.1. Arsenic-Induced Oxidative Stress

Being a metalloid, arsenic showed molecular toxicity by oxidative stress [155,156]. Arsenic induces the generation of free radicals and diminishes the activity of the antioxidant system in the body. In the Leydig and Sertoli cells of mice testes, exposure to sodium arsenite at concentrations of 50 and 1000 ppb for 24, 48, and 72 h caused cytotoxicity and impaired antioxidant systems [144]. In vitro cell cultures of rodent testes and epididymis treated with sodium arsenite at concentrations of 1, 10, 50, and 100 µM for 2 and 24 h increased ROS, TBARS, and sperm DNA damage, decreased catalase, peroxidase, and superoxide dismutase, and decreased serum testosterone [157]. Sodium arsenite at a dose of 10 mg/L through drinking water was shown to induce overexpression of SOD1, CAT, GSTK1, and MT1 in the testes and epididymis of rats [148].

### 2.2. Arsenic-Induced Apoptosis

**Figure 4:**
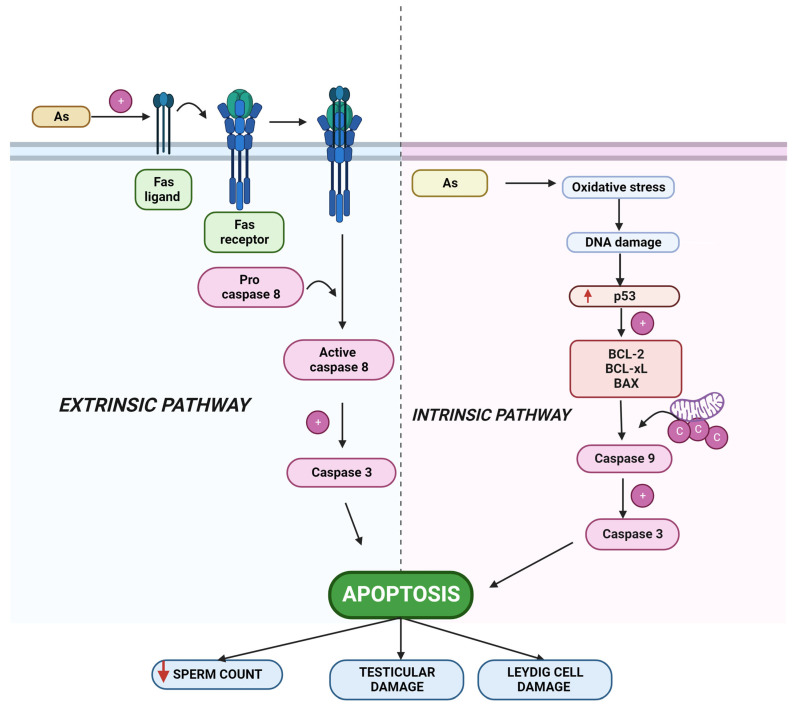
Diagram demonstrating how arsenic induces the intrinsic and extrinsic apoptotic pathways, which result in the death of apoptotic cells in the testes. As, arsenic, Bcl2, B-cell lymphoma 2; Bcl-xL, B-cell lymphoma-extra-large; BAX, Bcl-2-associated X protein.

Reduced sperm counts, increased caspase-3 activity, increased TUNEL-positive cells, changes in mRNA levels of Bax and Bcl-2 decreased serum testosterone levels, and downregulated expression of steroidogenic genes (LHR, StAR, and ABP) were observed in mice exposed to sodium arsenite and sulphur dioxide at doses of 5 mg/L and 5 mg/m^3^, respectively, through double distilled water for 60 days oral administration [158]. In mice, arsenic trioxide and antimony at doses of 4 and 15 mg/kg through intragastric administration for two months resulted in lower serum testosterone levels, fewer spermatogonia and sperm counts, and lower T-AOC, SOD, and MsrB 1 levels, as well as increased Beclin-1, Atg-5, LC3B/LC3A, caspase-8, Cytc, cleaved caspase-3, and p53 [159]. Figure 4 shows arsenic-induced apoptosis in the testis.

### 2.3. Arsenic-Induced Autophagy

Autophagy is a major cellular mechanism that allows cells to break down and reuse old cell parts, allowing them to operate more efficiently. Several studies have shown that arsenic-induced disruption in autophagy mechanisms causes a variety of diseases such as cancer, metabolic disorders, and reproductive toxicity [160,161]. Sodium arsenite exposure at concentrations of 3, 6, and 9 µM for 24 h resulted in the accumulation of autophagosomes with the upregulation of LC3β, Atg7, Beclin-1, and Vps34 autophagic markers expression in mouse testes Leydig tumour cell lines (MLTC-1) [152]. In the GC-1 spermatogonial (spg) cell line, arsenic trioxide at concentrations of 10 and 20 µM resulted in a drop in GSH and an increase in Malondialdehyde (MDA) levels, as well as elevation of ATG3, p62, LC3I, and LC3II mRNA expression, indicating mitochondrial dysfunction [154]. From five weeks before mating of parental mice and post-natal days up to adulthood, As trioxide exposure at concentrations of 0.2, 2, and 20 ppm in distilled water resulted in an increase in PI3K, Atg5, Atg12 gene expression and Beclin-1, LC-3I, II, and P62 protein expression in HPG axis tissues in F1 males [41]. In the testes of chickens, As trioxide at doses of 0.625, 1.25, and 2.5 mg/kg body weight for 30, 60, and 90 days elevated oxidative stress, exacerbated LC3-II, dynein, Beclin-1, ATG5, and ATG4B mediated autophagy, and triggered apoptosis [151]. Exposure of sodium arsenite to MLTC-1 cell lines at concentrations of 1, 2, and 4 mg/L for 48 h resulted in downregulation of Star, P450scc, P45-c17, and 17β-HSD genes and in contrast, increased mRNA and protein expression of 3β-HSD was observed [153]. Upregulation of H3K9me3 methyltransferase, Suv39h1, and downregulation of Jmjd2a demethylase were observed by sodium arsenite exposure at a dose of 1, 5, and 25 mg/L through drinking water for 6 months which led to steroidogenic gene repressions such as Lhr, StAR, P450scc, 3β-HSD, 17β-HSD, Cyp17a1, and Arom [147]. Additionally, sodium arsenite at the concentration of 5 and 50 ppm for 6 months reduced LHR, StAR, 3β-HSD, and 17β-HSD expressions and downregulated StAR, 17β-HSD, and Ddx3y mRNA levels in mice testes [145].

### 2.4. Arsenic-Induced Inflammation

Chickens exposed to copper sulphate and arsenic trioxide at the doses of 300 and 30 mg/kg through the feed for 4, 8, and 12 weeks showed an increase in mRNA levels of proinflammatory cytokines and inflammatory factors. They showed an increase in mRNA and protein levels of Hsp60, Hsp70, and Hsp90 as a protective effect from inflammatory damage [150]. NF-kβ, TNF-α, i-NOS, COX-2, and PTGEs mRNA overexpression were observed in chickens at dietary doses of 7.5, 15, and 30 mg/kg for 30, 60, and 90 days, whereas Hsp70 and Hsp90 mRNA expressions were also raised as a cell defence mechanism against As exposure [149]. Ferroptosis has been reported as one of the toxicity pathways of arsenic [162]. Sodium arsenite exposure through drinking water induced ferroptosis signalling in the testis of mice and GC-2 spg cell lines at concentrations of 0.5, 5, and 50 ppm for six months [163].

## 3. Effects of Prenatal Exposure to Arsenic in Humans

A study on 1390 pregnant women in Wuhan, China who were exposed to As revealed a reduction in birth weight, birth length, and risk of SGA in newborns [164]. A case study conducted with Unexplained Recurrent Spontaneous Abortion (URSA) patients at Beijing Maternal and Child Health Care Hospital found an increased level of arsenic in the blood, which suggests that blood arsenic may increase the risk of URSA in women of childbearing age [165]. A cohort study of 205 pregnant women in Hanam province, Vietnam revealed that prenatal exposure to drinking water containing high levels of arsenic showed increased cord arsenic levels, 8-OHdG, 8-nitroguanine, DNA strand break, and MN frequency, illustrating the genotoxic effects of arsenic [157]. Low levels of arsenic exposure during pregnancy showed maternal and neonatal thyrotoxicity [166]. In-utero exposure to arsenic in 706 pregnant women has shown an increase in birth length, decreased head circumference, and reduced adiposity in infants. A cohort study in Mexico City revealed transplacental arsenic exposure had shown an increased risk of SGA and large-for-gestational-age (LGA) and enhanced maternal arsenic blood levels [167]. Systematic review and metanalysis of maternal arsenic exposure showed a decrease in the birth weight, head circumference, and birth length in which gestational exposure increased hypomethylated cytosines in active retrotransposons long interspersed nuclear elements (LINEs) and long terminal repeat (LTRs) [168].

### 3.1. Effects of Arsenic on the Male Reproductive System in Humans

**Figure 5:**
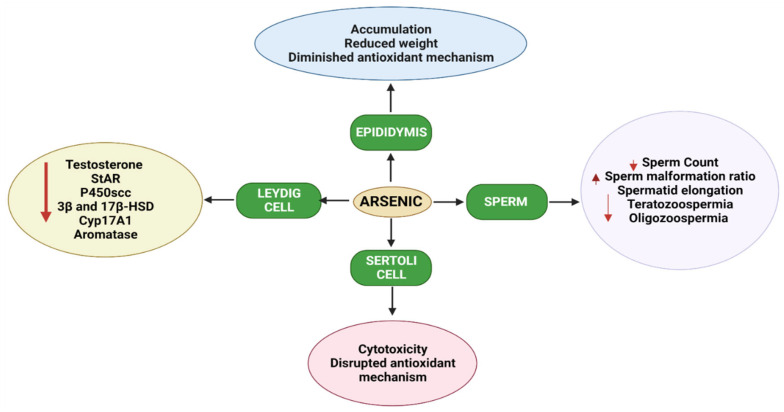
Diagram demonstrating how exposure to arsenic has a negative impact on Leydig’s and Sertoli cells, sperm, and epidydimis, and eventually damages the testis at the cellular and gene levels. StAR, steroidogenic acute regulatory protein; CYP17A1, Cytochrome P450 17A1; 17βHSD, 17-beta (β)-hydroxysteroid dehydrogenase.

A case-control study reported a positive correlation between urinary arsenic species with Unexplained Male Infertility (UMI) and a decrease in methylation of arsenic in 101 patients. Exposure to arsenic increased very low birth weight (VLBW) and preterm birth (PTB) in the people of Ohio, USA [169]. Prenatal exposure to sodium arsenite at a concentration of 85 ppm from 8 to 18 days showed transgenerational inheritance of impaired spermatogenesis phenotyping involving a decrease in the methylation status of Igf2, DMR2, and H19 DMR with a relative increase in mRNA and abnormal expression of Igf2 and H19 [170]. Arsenic exposure in 452 males was associated with increased urinary hormone excretion [171]. A cohort study of 127 male subjects in the hospitals of Nanjing Medical University, China showed the presence of urinary arsenic levels of inorganic arsenic (iAs), MMA, DMA, and arsenobetaine (AsB) which were correlated with male infertility risk [172]. Combined heavy metal exposure of arsenic and lead and cadmium has shown increased 8-OHdG, 8-isoPGF2α, and HNE-MA, which illustrated that higher urinary As, cadmium, and lead levels were associated with increased oxidative stress leading to alteration in semen quality [173]. A cohort study of 96 subjects in China revealed elevated levels of creatinine, arsenobetaine, DMA, MMA, arsenite, and arsenate were associated with poor semen quality [17]. In NJMU hospitals, a positive correlation was found between environmental arsenic exposure and male sexual dysfunction [171,172]. In conclusion, these findings suggest that As plays a critical role as a toxicant in the dysfunctions of male reproductive toxicity. The overall effect of arsenic on male reproductive cells is depicted in Figure 5.

### 3.2. Ameliorating Agents for Arsenic Toxicity

Phytonutrients are plant-produced natural substances or compounds. They have health-promoting bioactive effects via enhancing immunity and exerting antioxidant characteristics (shown in Table 4). The major molecular mechanisms involved in arsenic toxicity were oxidative stress, apoptosis, autophagy, and inflammation. Many phytonutrients showed protective benefits against arsenic-induced reproductive toxicity. A study using *Chlorophytum borivilianum* showed a reduction in As-induced lipid peroxidation, acid and alkaline phosphatase, and cholesterol in mouse Leydig and Sertoli cells [174]. Further, α-lipoic acid (LA) at a dose of 70 mg/kg was effective against arsenic-induced testicular toxicity, and results showed that LA decreased the mRNA expression of caspase-3 [175]. A total of 30 days of treatment with the formulated high-protein diet (FHPD), containing 15% casein and 7% pea protein in arsenic-exposed rats, inhibited lipid peroxidation, and the findings inferred that FHPD may have chelation properties and improve urinary arsenic excretion by increasing methylation [176]. The oral administration of *Pulsatilla nigricans* at a dose of 35 mg/kg for 90 days increased sperm maturation by increasing sorbitol dehydrogenase levels, as well as increasing GSH, SOD, and CAT levels and decreasing LDH and γ-GT levels in the testis [177]. The activation of NF-kβ and expression of i-NOS, COX-2, TNF-α, and IL after As exposure was inhibited by giving ellagic acid at doses of 10 and 30 mg/kg orally for 14 days in rat testis [178]. The expressions of Nfe2l2, StAR, and Ppargc1a, sperm morphology, and antioxidant levels in mice were restored after 40 days of oral administration of ellagic acid and ferulic acid at doses of 50 mg/kg each [179]. Lutein treatment at a dose of 40 mg/kg given orally for five weeks increased mRNA expression of Nrf-2 downstream genes (HO-1, GST, and NQO1) in response to As exposure [180]. Proanthocyanidin, grape seed extract, given orally at doses of 100 and 200 mg/kg for 5 weeks, boosted T-AOC, Nrf-2 expression, GSH, and SOD activity, while decreasing MDA and 8-OHdG levels [181]. Green tea component, epigallocatechin-3-gallate (EGCG) at a dose of 20 mg/kg intraperitoneally for 40 days restored sperm kinetic characteristics, structural membrane integrity (SMI), and functional membrane integrity (FMI). It potentiated the activity of the Nrf-2 pathway and the production of different antioxidants [182]. Treatment with N-Acetyl Cysteine (NAC) at a concentration of 40 ppm for five weeks improved sperm motility, morphology, and weight of seminal vesicles. It boosted the GSH concentration and activity by acting as a precursor for GSH [183]. Further, NAC given intraperitoneally at a dose of 75 mg/kg for 40 days enhanced sperm parameters, 3β-HSD, 17β-HSD, and SOD and Catalase activities against As exposure by chelating As [184]. Polydatin treatment at doses of 50, 100, and 200 mg/kg for 60 days with oral administration restored As-induced sperm destruction by raising SOD and CAT levels in the testicular tissue of rats [185]. Melatonin at a dose of 25 mg/kg for 30 days repaired As-induced damage to the mean seminiferous tubular diameter (MSTD), mean testicular biopsy scores (MTBS), and proliferating cell nuclear antigen (PCNA) in rats [182]. Pistia stratiotes treatment at the dose of 100 mg/kg for 14 days of oral administration had shown protective action against As-induced sperm damage by restoring sperm motility, viability, count, and semen volume in rats [186]. Oral administration of chlorogenic acid at doses of 100 and 200 mg/kg for four weeks in the testicular tissue of mice exhibited antioxidant, anti-inflammatory, anti-apoptotic, and Nrf-2 activation against As toxic effects [185]. Oral therapy with selenium and diphenyl diselenide (DPDS) at a dose of 2.5 mg/kg for 45 days reduced inflammation, myeloperoxidase, NO, TNF-α, and IL-1 activity in the testes and epididymis of As-exposed rats [187]. Sodium arsenite-induced reproductive damage in hamsters was reversed by α- tocopherol succinate (α-TOS) and sodium selenite (SS) at a dose of 6 mg/kg and 0.025 mg/kg from the 1st day of gestation till delivery showed decreased teratogenic effects. In contrast, SS was shown to increase the methylation process, whereas α-TOS enhanced antioxidant activity [57].

### 3.3. Other Phytonutrients That Promote Male Fertility

Coenzyme-Q10 is a naturally occurring hydrophobic molecule with potent antioxidant properties that have also been found to inhibit the generation of TNF-α, NO, and NF-Kβ as well as the activation of apoptosis in rats when given at a dose of 10 mg/kg intraperitoneally for five days [189,199]. Oral administration of quercetin at a dose of 50 mg/kg for 15 days improved serum testosterone levels, restored testicular architecture, and reduced TUNEL-positive cells [191], whereas exposure for 49 days showed powerful chelating and antioxidant properties to defend against lipid peroxidation, which recovered daily sperm production, sperm count, and reversed sperm DNA damage in the epididymis and testis of rats [192,193]. Oral administration of *Withania sominifera* at a dose of 100 mg/kg improved male libido by restoring spermatogenesis, sperm morphology, and testicular architecture, as well as boosting blood LH and testosterone levels [194]. Combined oral treatment of zinc chloride and vitamin C at doses of 20 mg/kg and 100 mg/kg restored sperm morphology, count, and seminiferous tubule diameter. Zinc works as a cofactor for SOD and can chelate As, demonstrating its antioxidant properties in rats, whereas Vitamin C at a dose of 200 mg/kg increased testosterone, FSH, and LH and improved histopathological lesions in Teddy goat bucks [195]. Oral administration of *Alchornea cordifolia* at 100 µg/kg for 30 days raised testosterone, FSH, spermatozoa count, and motility, as well as the expression of androgen receptor binding protein, and anti-apoptotic B-cell lymphoma-2 in the testis of rats [196]. Treatment with 200 mg/kg Vitamin E for 84 days increased spermatogenesis and restored blood LH, FSH, and testosterone levels [167], and improved semen quality in Teddy goat buck testicular tissue [200]. Oral administration of D-ribose-L-Cysteine at doses of 10 and 30 mg/kg over 28 days restored sperm count, motility, viability, LH, FSH, testosterone, and CAT, SOD, and GSH activities in rat testes [197]. In a recent study, nano vitamin C (NVC) was found to ameliorate arsenic-induced changes in testicular and sperm parameters. Significant increases in GPx, SOD, and CAT, as well as elevated serum levels of LH, FSH, and testosterone, were found in arsenic-treated male rats receiving 200 mg/kg of NVC [201]. Similarly, broccoli extract (300 mg/kg) treatment resulted in a significant reduction in oxidative stress and a rise in SOD, GPx, and total antioxidant capacity (TAC); nevertheless, a decrease in malondialdehyde (MDA) content was seen in the group compared to the As group. These results suggest that broccoli extracts are highly effective at reducing liver and kidney damage as well as improving haematological and biochemical variables in arsenic poisoning circumstances [202]. These findings clearly suggest that phytonutrients play a key role in combating arsenic-induced toxicity. However, further research needs to be conducted to explore the untapped potential of phytonutrients in ameliorating arsenic chronic toxicity.

## 4. Conclusions and Future Directions

Arsenic is a widespread metalloid that exerts a detrimental effect on male reproductive health in both animals and humans. Major sources of arsenic contamination include drinking water, food, and industrial waste. Arsenic impaired sperm quality, decreased sperm count, sperm viability, induced spermatozoa apoptosis, and damaged testicular and epididymal tissues and sperm DNA. Arsenic exposure also affects testosterone levels by impairing FSH and LH levels, affecting spermatogenesis, and producing male sterility in both animals and humans. Additionally, arsenic has shown transplacental developmental toxicity, which increases the chance of SGA and causes fetal abnormalities such as lower birth weight and preterm birth. However, the reproductive toxicity of arsenic is poorly understood, and the molecular mechanisms of arsenic-induced male reproductive toxicity remain unclear. Inflammatory response, oxidative stress, autophagy, and apoptosis are some of the possible arsenic-mediated toxicity pathways. On the other hand, phytonutrients have an essential protective function against arsenic-induced male reproductive toxicity. Phytonutrients, plant-based bioactive components, improve male fertility by boosting immunity and exerting antioxidant properties that diminish the oxidative and inflammatory stress generated by arsenic in reproductive cells. However, future research is needed to identify more phytoconstituents and understand their molecular mechanisms to completely mitigate and/or reverse the deleterious effects of arsenic poisoning. Furthermore, governments must take stringent measures to reduce arsenic levels in drinking and groundwater, food, and industrial effluents, thereby lowering human and animal exposure levels.

## Figures and Tables

**Table 1:** Compilation of research studies showing the effect of arsenic exposures among diverse demographics in various parts of the world.

S. No	Countries	Population (Subjects)	Sample Size	Sample	Detected Levels of Arsenic	Reference
1	Argentina	Children (3–15 years)	101	Hair, Urine	110–1311 μg/kg	[73]
2	Montevideo, Uruguay	Children (5–8 years)	328	Drinking water	9.9 μg/L	[74]
					0.45 μg/L	
3	Shaanxi province, China	Adults	96	Drinking water	4.52 µg/L	[75]
				Indoor air	0.03 mg/m^3^	
				Soil	14930 mg/kg	
4	Inner Mongolia	Adults	96	Drinking water	144.71 µg/L	
				Soil	10190 mg/kg	
5	Villages of Pakistan	Children (≤16 years)	223	Ground water	15. 63 µg/kg/day (arsenate) 0.09 µg/kg/day (arsenite)	[76]
		Adult			15.07 µg/kg/day (arsenate) 0.26 µg/kg/day (arsenite)	
6	Indae metal mine area	Residents (mean age of 66.8 years)	50	Urine	Arsenite (1.45 µg/L), arsenate (0.74 µg/L), MMA (2.43 µg/L), DMA (27.63 µg/L), and arsenobetaine (88.62 µg/L)	[77]
7	Spain	Children (4–5 years)	400	Urine	2.74–7.54 µg/L	[78]

**Table 2:** Evidence from research findings indicates the presence of arsenic in grains, vegetables, dairy products, and meat which increases the risk of cancer.

S. No	Country	Sample (s)	Arsenic Levels	Reference
1	Kolkata, India	Rice, grain, and vegetable	76 µg/kg and 41.4 µg/kg	[79]
2	UK	Rice	130 μg/kg	[80]
3	Kunming, China	Rice	3520 µg/kg	[81]
4	Japan	Rice, hijiki	19 µg/kg and 59 µg/kg	[82]
5	Pakistan	Raw rice	92.5 ± 41.88 μg/kg	[83]
		Cooked rice	79.21 ± 76.42 μg/kg	
		Wheat	116.38 ± 51.38 μg/kg	
6	West Bengal, India	Boro and Aman rice	194 μg/kg and 156 μg/kg	[79]
		Arum and radish	780 and 674 μg/kg	
		Urine	154–276 µg/L	
7	Ngari area, Western Tibet	Barley	180 ± 210 µg/kg	[84]
		Vegetable	400 ± 570 µg/kg,	
		Meat	210 ± 160 µg/kg	
		Dairy products	180 ± 80 µg/kg	
		Daily intake	3600 µg/kg	
8	Kurdistan province, Iran	Sheep meat	230 ± 140 µg/kg	[85]
		Beef meat	200 ± 90 µg/kg	
		Turkey meat	880 ± 210 µg/kg	
		Ostrich meat	850 ± 130 µg/kg	

**Table 4:** The table summarises preclinical findings and interventions for arsenic exposure induced male infertility.

S. No	Experimental Model	Phytonutrient	Treatment	Route of Administration	Duration	Organ/Tissue	Observations	Reference
**1**	Mice	*C. borivilianum*	Sodium arsenite 4 mg/kg bw, *C. borivilianum* 100, 200, 400, and 800 mg/kg bw	Oral	30 days	Testes	Decreased acid phosphatase, alkaline phosphatase, and cholesterol levels.	[174]
**2**	Mice	Ellagic acid and Ferulic acid	Sodium arsenate dibasic heptahydrate–200 ppm and Ellagic acid 50mg/kg and Ferulic acid 50 mg/kg	Oral	40 days	Testes and sperm	Restored sperm morphology characteristics, anti-oxidant levels, enhanced expression of Nfe2l2 and StAR, reduced Ppargc1a.	[179]
**3**	Mice	Lutein	As trioxide–5 mg/kg bw and Lutein 40 mg/kg bw	Oral	35 days	Testes and sperm	Increased sperm count. Enhanced expression of Nrf-2 and downstream genes (HO-1, GST and NQO1).	[180]
**4**	Mice	Grape seed proanthocyanidin extract	As trioxide–4 mg/kg bw, grape seed proanthocyanidin extract 100 and 200 mg/kg bw	Oral	35 days	Testes	Reduction in MDA, 8-OHdG, increased T-AOC and activities of GSH and SOD. Elevated expression of genes related to Nrf-2 signalling pathway.	[181]
**5**	Mice	Epigallocatechin (EGCG)	Sodium arsenite heptahydrate–200 ppm, Epigallocatechin 3–20 mg/kg bw	Intraperitoneal	40 days	Testes	Restored sperm morphology, SMI, FMI, serum testosterone, antioxidant system.	[183]
**6**	Mice	N-acetyl cysteine (NAC)	Arsenic trioxide–0.3 and 3 mg/kg bw	Subcutaneous	35 days	Sperm and Seminal vesicle	Restored seminal vesicle weight, sperm motility, daily sperm production.	[188]
			NAC–40mM	Oral				
**7**	Mice	N-acetyl cysteine (NAC)	Sodium arsenite–4 ppm, NAC–75 mg/kg bw	Intraperitoneal	40 days	Testes	Restored weight of testes, epididymis, seminal vesicles and ventral prostate and increase in sperm parameters, 3βHSD, 17βHSD, and SOD catalase activities.	[184]
**8**	Mice	Chlorogenic acid (CGA)	Sodium arsenite–5 mg/kg and CGA–100 and 200 mg/kg bw	Oral	28 days	Testes	Antioxidant, anti-inflammatory, anti-apoptotic, and activates Nrf-2 pathway.	[185]
**9**	Rat	α-lipoic acid	Sodium arsenite–25 mg/L and α-lipoic acid–70 mg/kg bw	Intraperitoneal	56 days	Testes	Restoration of testicular architecture, testicular sperm production, 3β and 17β-HSDs.	[175]
**10**	Rat	FHPD	As trioxide–3 mg/kg bw 7% pea and 15% casein were added.	Oral	30 days	Testes and sperm	Restored the number of total motile spermatozoa, maintains testosterone levels, restored anti-oxidant levels.	[176]
**11**	Rat	Ellagic acid	Sodium arsenite 10 mg/kg bw Ellagic acid 10 and 30mg/kg bw	Oral	14 days	Testes	Restored serum testosterone, testicular anti-oxidant level and structural changes.	[178]
**12**	Rat	Polydatin	As trioxide–100 mg/L, Polydatin–50,100 and 200 mg/kg bw	Oral	60 days	Sperm	Enhanced sperm membrane integrity, sperm morphology, enhanced epididymal sperm motility.	[182]
**13**	Rat	Melatonin	Sodium arsenite–5 mg/kg bw and Melatonin–25 mg/kg bw	Oral	30 days	Testes and sperm	Improved body weight, testicular weight, reduced the TUNEL positive germ cells enhanced PCNA index.	[189]
**14**	Rat	Co-enzyme Q10	Sodium arsenite–10 mg/kg bw Co-enzyme Q10–10 mg/kg	Intraperitoneal	5 days	Testes	Restored serum testosterone, restored anti-oxidant, TNF-α, NO, restored testis architecture and active spermatogenesis, reduction in i-NOS, NF-kβ, Fas ligand and caspase-3 in testis.	[190]
**15**	Rat	Quercetin	Sodium arsenite–10 mg/kg bw Quercetin–50 mg/kg bw	Oral	15 days	Testes	Restored testicular architecture, reduced TUNEL positive cells.	[191]
**16**	Rat	Quercetin	Sodium arsenite–50 ppm Quercetin–50 mg/kg bw	Oral	49 days	Epididymis	Recovered daily sperm production, sperm count, and reversed sperm DNA damage.	[192]
**17**	Rat	Quercetin	Sodium arsenite–50 ppm Quercetin–50 mg/kg bw	Oral	49 days	Testes	Restored GSH, CAT, SOD, POD, TBARS, and testosterone.	[193]
**18**	Rat	Withania somnifera	Sodium arsenite–8 mg/kg bw Withania somnifera–100 mg/kg bw	Oral	30 days	Testes and sperm	Restored sperm morphology characteristics, serum testosterone, decreased LPO, restored spermatogenesis, and testicular architecture.	[194]
**19**	Rat	Zinc chloride and Vitamin C	Sodium arsenite–5 mg/kg bw Zinc chloride–20 mg/kg bw Vitamin C–100 mg/kg bw	Oral	60 days	Testes and epididymis	Restored sperm morphology, count, and seminiferous tubule diameter.	[195]
**20**	Rat	Pistia stratiotes	Pistia stratiotes–100 mg/kg Sodium arsenite–2.5 mg/kg	Oral	14 days	Sperm	Restored sperm motility, viability, count, and semen volume.	[186]
**21**	Rat	Alchornea cordifolia	Sodium arsenite–7 mg/kg bw Alchornea cordifolia–100 µg/kg bw	Oral	30 days	Testes	Enhanced testosterone, FSH, spermatozoa count, and motility. Expression of Androgen receptor binding protein and anti-apoptotic B-cell lymphoma-2.	[196]
**22**	Rat	D-Ribose-L-Cysteine	Sodium arsenate–8 mg/kg bw D-Ribose-L-Cysteine–10 and 30 mg/kg bw	Oral	28 days	Testes	Restored sperm count, motility and viability, LH, FSH, and testosterone and CAT, SOD, GSH.	[197]
**23**	Rat	Selenium and Diphenyl diselenide (DPDS)	Sodium arsenite–60 µg/L Selenium–0.25 mg/kg bw and DPDS–2.5 mg/kg bw	Oral	45 days	Testes and epididymis	Suppressed inflammation, myeloperoxidase activity, NO, TNF-α, and IL-1.	[187]
**24**	Teddy goat buck	Vitamin E	Sodium arsenite–5 mg/kg bw Vitamin E–200 mg/kg bw	Oral	84 days	Testes	Enhanced spermatogenesis, restored germinal epithelium, and enhanced testosterone, FSH, LH, and ameliorated histopathological lesions. Serum LH, FSH, and testosterone were restored and improved semen quality.	[167]
**25**	Hamster	α- tocopherol succinate (α-TOS) and sodium selenite (SS)	Sodium arsenite 100 ppm α-TOS–6 mg/kg bw and SS–0.025mg/kg bw	Oral	22 days	Placenta and fetus	Decreased teratogenic effects. SS increased methylation process and α-TOS enhanced antioxidant activity.	[198]

## Data Availability

Not applicable.

## Abbreviations

SGA	Small-for-gestational age
WHO	World Health Organization
EPA	Environmental Protection Agency
IARC	International Agency for Research on Cancer
AS_2_O_3_	Arsenic trioxide
CML	Chronic myelogenous leukaemia
APL	Acute promyelocytic leukaemia
TBARS	Thiobarbituric acid reactive substances
HPG axis	The hypothalamic–pituitary–gonadal axis
StAR	The steroidogenic acute regulatory
3β-HSD	3β-Hydroxysteroid dehydrogenase
GIT	Gastrointestinal tract
AQP	Aquaglyceroporins
MFS	Major facilitator superfamily
ABC	ATP-binding cassette
As3MT	Arsenite methyltransferase
MMA	Monomethylarsonic acid
DMA	Dimethylarsinic acid
As(V)	Inorganic pentavalent arsenic
As(III)	Inorganic trivalent arsenic
MMA(V)	Methyl arsonate
DMA(V)	Dimethyl arsenate
GSTO	Glutathione S-transferase omega
GSSG	Glutathione disulfide
As-GSH	Arsenic glutathione
AsS	Arsenic sulphide
AsB	Arsenobetaine
ROS	Reactive oxygen species
ICR	Institute of Cancer Research
CC3	Cleaved caspase 3
CYP17A1	Cytochrome P450 17A1
DHT	Dihydrotestosterone
SD	Sprague Dawley
MLTC-1	Mouse testes Leydig tumor cell lines
MDA	Malondialdehyde
NAPDH	Nicotinamide adenine dinucleotide phosphate (NADP+)
NOX	NAPDH oxidase
GPx	Glutathione peroxidase
SOD	Supeoxide dismutase
CAT	Catalase
H2O2	Hydrogen peroxide
LOO	Lipid peroxy radical
UMI	Unexplained male infertility
VLBW	Very low birth weight
PTB	Preterm birth
EGCG	Epigallocatechin-3-gallate
SMI	Structural membrane integrity
FMI	Functional membrane integrity
NAC	N-Acetyl Cysteine
MSTD	Mean seminiferous tubular diameter
MTBS	Mean testicular biopsy scores
PCNA	Proliferating cell nuclear antigen
DPDS	Diphenyl diselenide
BTB	Bllod testicular border

## References

[1] Rossy K.M., Janusz C.A., Schwartz R.A. (2005). Cryptic Exposure to Arsenic. Indian J. Dermatol. Venereol. Leprol..

[2] Verma N., Rachamalla M., Sravan Kumar P., Dua K. (2023). Assessment and Impact of Metal Toxicity on Wildlife and Human Health.

[3] Mandal B.K., Suzuki K.T. (2002). Arsenic Round the World: A Review. Talanta.

[4] Antman K.H. (2001). Introduction: The History of Arsenic Trioxide in Cancer Therapy. Oncologist.

[5] Zhu J., Chen Z., Lallemand-Breitenbach V., de Thé H. (2002). Timeline: How Acute Promyelocytic Leukaemia Revived Arsenic. Nat. Rev. Cancer.

[6] Tamaki S., Frankenberger W.T. (1992). Environmental Biochemistry of Arsenic. Rev. Environ. Contam. Toxicol..

[7] Sears M.E., Kerr K.J., Bray R.I. (2012). Arsenic, Cadmium, Lead, and Mercury in Sweat: A Systematic Review. J. Environ. Public Health.

[8] Leist M., Casey R., Caridi D. (2000). The management of arsenic wastes: Problems and prospects. J. Hazard. Mater..

[9] Waxman S., Anderson K.C. (2001). History of the Development of Arsenic Derivatives in Cancer Therapy. Oncologist.

[10] Renu K., Madhyastha H., Madhyastha R., Maruyama M., Vinayagam S., Gopalakrishnan A.V. (2018). Review on molecular and biochemical insights of arsenic-mediated male reproductive toxicity. Life Sci..

[11] Kim Y.-J., Kim J.-M. (2015). Arsenic Toxicity in Male Reproduction and Development. Dev. Reprod..

[12] Chen Q.Y., Costa M. (2021). Arsenic: A Global Environmental Challenge. Annu. Rev. Pharmacol. Toxicol..

[13] Abdul K.S.M., Jayasinghe S.S., Chandana E.P.S., Jayasumana C., De Silva P.M.C.S. (2015). Arsenic and human health effects: A review. Environ. Toxicol. Pharmacol..

[14] Chung J.-Y., Yu S.-D., Hong Y.-S. (2014). Environmental Source of Arsenic Exposure. J. Prev. Med. Public Health.

[15] Oremland R.S., Stolz J.F. (2003). The Ecology of Arsenic. Science.

[16] Ramos A.T.D.A., Diamante M.A.S., Lamas C.D.A., Dolder H., Predes F.D.S. (2017). Morphological and morphometrical changes on adult Wistar rat testis caused by chronic sodium arsenite exposure. Environ. Sci. Pollut. Res..

[17] Xu W., Bao H., Liu F., Liu L., Zhu Y.-G., She J., Dong S., Cai M., Li L., Li C. (2012). Environmental exposure to arsenic may reduce human semen quality: Associations derived from a Chinese cross-sectional study. Environ. Health.

[18] Quansah R., Armah F., Essumang D.K., Luginaah I., Clarke E., Marfoh K., Cobbina S.J., Nketiah-Amponsah E., Namujju P.B., Obiri S. (2015). Association of Arsenic with Adverse Pregnancy Outcomes/Infant Mortality: A Systematic Review and Meta-Analysis. Environ. Health Perspect..

[19] Podgorski J., Berg M. (2020). Global threat of arsenic in groundwater. Science.

[20] Vidosavljevic M., Puntaric D., Gvozdic V., Vidosavljevic D., Juric D., Begovic L. (2022). Assessment of Arsenic in Hair of the Inhabitants of East Croatia—Relationship to Arsenic Concentrations in Drinking Water. Water.

[21] Rahman M., Tondel M., Ahmad S.A., Axelson O. (1998). Diabetes Mellitus Associated with Arsenic Exposure in Bangladesh. Am. J. Epidemiol..

[22] Kile M.L., Christiani D.C. (2008). Environmental Arsenic Exposure and Diabetes. JAMA-J. Am. Med. Assoc..

[23] Hopenhayn C., Ferreccio C., Browning S.R., Huang B., Peralta C., Gibb H., Hertz-Picciotto I. (2013). Arsenic Exposure from Drinking Water and Birth Weight. Epidemiology.

[24] Bardach A.E., Ciapponi A., Soto N., Chaparro M.R., Calderon M., Briatore A., Cadoppi N., Tassara R., Litter M.I. (2015). Epidemiology of Chronic Disease Related to Arsenic in Argentina: A Systematic Review. Sci. Total Environ..

[25] Smith A.H., Lopipero P.A., Bates M.N., Steinmaus C.M. (2002). Arsenic Epidemiology and Drinking Water Standards. Science.

[26] Rahman M.M., Chowdhury U.K., Mukherjee S.C., Mondal B.K., Paul K., Lodh D., Biswas B.K., Chanda C.R., Basu G.K., Saha K.C. (2001). Chronic Arsenic Toxicity in Bangladesh and West Bengal, India—A Review and Commentary. J. Toxicol. Clin. Toxicol..

[27] Mazumder D.N.G. (2003). Chronic Arsenic Toxicity: Clinical Features, Epidemiology, and Treatment: Experience in West Bengal. J. Environ. Sci. Health Part A Tox Hazard. Subst. Environ. Eng..

[28] Singh A., Ghosh A.K. (2014). Groundwater Arsenic Contamination and Its Implications: A Case Study of Shahpur Block of Bhojpur District, Bihar. Int. J. Mod. Eng. Res..

[29] Chakraborti D., Singh S.K., Rahman M.M., Dutta R.N., Mukherjee S.C., Pati S., Kar P.B. (2018). Groundwater Arsenic Contamination in the Ganga River Basin: A Future Health Danger. Int. J. Environ. Res. Public Heal..

[30] Yang H.-C., Rosen B.P. (2016). New mechanisms of bacterial arsenic resistance. Biomed. J..

[31] Palma-Lara I., Martínez-Castillo M., Quintana-Pérez J., Arellano-Mendoza M., Tamay-Cach F., Valenzuela-Limón O., García-Montalvo E., Hernández-Zavala A. (2020). Arsenic exposure: A public health problem leading to several cancers. Regul. Toxicol. Pharmacol..

[32] Wang W., Cheng S., Zhang D. (2014). Association of inorganic arsenic exposure with liver cancer mortality: A meta-analysis. Environ. Res..

[33] Cardoso A.P.F., Udoh K.T., States J.C. (2020). Arsenic-induced changes in miRNA expression in cancer and other diseases. Toxicol. Appl. Pharmacol..

[34] Cohen S.M., Chowdhury A., Arnold L.L. (2016). Inorganic arsenic: A non-genotoxic carcinogen. J. Environ. Sci..

[35] Martinez V.D., Vucic E.A., Becker-Santos D.D., Gil L., Lam W.L. (2011). Arsenic Exposure and the Induction of Human Cancers. J. Toxicol..

[36] Darbandi M.P., Taheri J. (2018). Using Sulfur-Containing minerals in medicine: Iranian traditional documents and modern pharmaceutical terminology. Earth Sci. Hist..

[37] Ommati M., Heidari R., Manthari R.K., Chiranjeevi S.T., Niu R., Sun Z., Sabouri S., Zamiri M., Zaker L., Yuan J. (2019). Paternal exposure to arsenic resulted in oxidative stress, autophagy, and mitochondrial impairments in the HPG axis of pubertal male offspring. Chemosphere.

[38] Leu L., Mohassel L. (2009). Arsenic Trioxide as First-Line Treatment for Acute Promyelocytic Leukemia. Am. J. Health-Syst. Pharm..

[39] Ratnaike R.N. (2003). Acute and chronic arsenic toxicity. Postgrad. Med. J..

[40] Calabrese E.J., Baldwin L.A. (2003). Inorganics and Hormesis. Crit. Rev. Toxicol..

[41] Ommati M.M., Manthari R.K., Tikka C., Niu R., Sun Z., Sabouri S., Zamiri M.J., Ahmadi H.N., Ghaffari H., Heidari R. (2020). Arsenic-induced autophagic alterations and mitochondrial impairments in HPG-S axis of mature male mice offspring (F1-generation): A persistent toxicity study. Toxicol. Lett..

[42] Ommati M.M., Shi X., Li H., Zamiri M.J., Farshad O., Jamshidzadeh A., Heidari R., Ghaffari H., Zaker L., Sabouri S. (2020). The mechanisms of arsenic-induced ovotoxicity, ultrastructural alterations, and autophagic related paths: An enduring developmental study in folliculogenesis of mice. Ecotoxicol. Environ. Saf..

[43] Dastgiri S., Mosaferi M., Fizi M.A.H., Olfati N., Zolali S., Pouladi N., Azarfam P. (2010). Arsenic Exposure, Dermatological Lesions, Hypertension, and Chromosomal Abnormalities among People in a Rural Community of Northwest Iran. J. Health Popul. Nutr..

[44] Maloney M.E. (1996). Arsenic in Dermatology. Dermatol. Surg..

[45] Thakur M., Rachamalla M., Niyogi S., Datusalia A.K., Flora S.J.S. (2021). Molecular Mechanism of Arsenic-Induced Neurotoxicity including Neuronal Dysfunctions. Int. J. Mol. Sci..

[46] Duker A.A., Carranza E.J., Hale M. (2005). Arsenic geochemistry and health. Environ. Int..

[47] Kannan G.M., Tripathi N., Dube S.N., Gupta M., Flora S. (2001). Toxic Effects of Arsenic (III) on Some Hematopoietic and Central Nervous System Variables in Rats and Guinea Pigs. J. Toxicol. Clin. Toxicol..

[48] Zhao H., Wang Y., Shao Y., Liu J., Wang S., Xing M. (2018). Oxidative stress-induced skeletal muscle injury involves in NF-κB/p53-activated immunosuppression and apoptosis response in copper (II) or/and arsenite-exposed chicken. Chemosphere.

[49] Bjørklund G., Aaseth J., Chirumbolo S., Urbina M.A., Uddin R. (2017). Effects of arsenic toxicity beyond epigenetic modifications. Environ. Geochem. Health.

[50] Hong Y.-S., Song K.-H., Chung J.-Y. (2014). Health Effects of Chronic Arsenic Exposure. J. Prev. Med. Public Health.

[51] Hosseinzadeh A., Houshmand G., Goudarzi M., Sezavar S.H., Mehrzadi S., Mansouri E., Kalantar M. (2018). Ameliorative effect of gallic acid on sodium arsenite-induced spleno-, cardio- and hemato-toxicity in rats. Life Sci..

[52] Kleiman N.J., Quinn A.M., Fields K.G., Slavkovich V., Graziano J.H. (2016). Arsenite accumulation in the mouse eye. J. Toxicol. Environ. Health Part A.

[53] Cui X., Okayasu R. (2008). Arsenic accumulation, elimination, and interaction with copper, zinc and manganese in liver and kidney of rats. Food Chem. Toxicol..

[54] Bae J., Jang Y., Kim H., Mahato K., Schaecher C., Kim I.M., Kim E., Ro S.-H. (2019). Arsenite exposure suppresses adipogenesis, mitochondrial biogenesis and thermogenesis via autophagy inhibition in brown adipose tissue. Sci. Rep..

[55] Martinez E.J., Kolb B.L., Bell A., Savage D.D., Allan A.M. (2008). Moderate perinatal arsenic exposure alters neuroendocrine markers associated with depression and increases depressive-like behaviors in adult mouse offspring. NeuroToxicology.

[56] Pakzad D., Akbari V., Sepand M.R., Aliomrani M. (2021). Risk of neurodegenerative disease due to tau phosphorylation changes and arsenic exposure via drinking water. Toxicol. Res..

[57] Rattan S., Zhou C., Chiang C., Mahalingam S., Brehm E., Flaws J.A. (2017). Exposure to endocrine disruptors during adulthood: Consequences for female fertility. J. Endocrinol..

[58] Wang A., Holladay S.D., Wolf D.C., Ahmed S.A., Robertson J.L. (2006). Reproductive and Developmental Toxicity of Arsenic in Rodents: A Review. Int. J. Toxicol..

[59] Biswas R., Poddar S., Mukherjee A. (2007). Investigation on the Genotoxic Effects of Long-Term Administration of Sodium Arsenite in Bone Marrow and Testicular Cells In Vivo Using the Comet Assay. J. Environ. Pathol. Toxicol. Oncol..

[60] Jana K., Jana S., Samanta P.K. (2006). Effects of chronic exposure to sodium arsenite on hypothalamo-pituitary-testicular activities in adult rats: Possible an estrogenic mode of action. Reprod. Biol. Endocrinol..

[61] Tikka C., Manthari R.K., Ommati M.M., Niu R., Sun Z., Zhang J., Wang J. (2020). Immune disruption occurs through altered gut microbiome and NOD2 in arsenic induced mice: Correlation with colon cancer markers. Chemosphere.

[62] Wu H., Wu R., Chen X., Geng H., Hu Y., Gao L., Fu J., Pi J., Xu Y. (2022). Developmental arsenic exposure induces dysbiosis of gut microbiota and disruption of plasma metabolites in mice. Toxicol. Appl. Pharmacol..

[63] Yan X., Chen X., Tian X., Qiu Y., Wang J., Yu G., Dong N., Feng J., Xie J., Nalesnik M. (2021). Co-exposure to inorganic arsenic and fluoride prominently disrupts gut microbiota equilibrium and induces adverse cardiovascular effects in offspring rats. Sci. Total Environ..

[64] Zhang T., Sun P., Geng Q., Fan H., Gong Y., Hu Y., Shan L., Sun Y., Shen W., Zhou Y. (2021). Disrupted spermatogenesis in a metabolic syndrome model: The role of vitamin A metabolism in the gut–testis axis. Gut.

[65] Zhao Q., Huang J.-F., Cheng Y., Dai M.-Y., Zhu W.-F., Yang X.-W., Gonzalez F.J., Li F. (2021). Polyamine metabolism links gut microbiota and testicular dysfunction. Microbiome.

[66] Jangra A., Verma M., Kumar D., Chandrika C., Rachamalla M., Dey A., Dua K., Jha S.K., Ojha S., Alexiou A. (2022). Targeting Endoplasmic Reticulum Stress using Natural Products in Neurological Disorders. Neurosci. Biobehav. Rev..

[67] Puppala E.R., Jain S., Saha P., Rachamalla M., Np S., Yalamarthi S.S., Abubakar, Chaudhary A., Chamundeswari D., Usn M. (2022). Perillyl alcohol attenuates rheumatoid arthritis via regulating TLR4/NF-κB and Keap1/Nrf2 signaling pathways: A comprehensive study onin-vitro and in-vivo experimental models. Phytomedicine.

[68] Zargari F., Rahaman S., KazemPour R., Hajirostamlou M. (2022). Arsenic, Oxidative Stress and Reproductive System. J. Xenobiotics.

[69] Adewoyin M., Ibrahim M., Roszaman R., Isa M.L.M., Alewi N.A.M., Rafa A.A.A., Anuar M.N.N. (2017). Male Infertility: The Effect of Natural Antioxidants and Phytocompounds on Seminal Oxidative Stress. Diseases.

[70] Khair A., Awal M.A., Hoque M.N., Talukder A.K., Das Z.C., Rao D.R., Shamsuddin M. (2021). Spirulina ameliorates arsenic induced reproductive toxicity in male rats. Anim. Reprod..

[71] Kumarathilaka P., Seneweera S., Ok Y.S., Meharg A., Bundschuh J. (2019). Arsenic in cooked rice foods: Assessing health risks and mitigation options. Environ. Int..

[72] Taylor V., Goodale B., Raab A., Schwerdtle T., Reimer K., Conklin S., Karagas M.R., Francesconi K.A. (2017). Human exposure to organic arsenic species from seafood. Sci. Total. Environ..

[73] Calatayud M., Farias S.S., de Paredes G.S., Olivera M., Carreras N.., Giménez M.C., Devesa V., Vélez D. (2019). Arsenic exposure of child populations in Northern Argentina. Sci. Total Environ..

[74] Kordas K., Queirolo E.I., Mañay N., Peregalli F., Hsiao P.Y., Lu Y., Vahter M. (2016). Low-level arsenic exposure: Nutritional and dietary predictors in first-grade Uruguayan children. Environ. Res..

[75] Wei B., Yu J., Kong C., Li H., Yang L., Guo Z., Cui N., Xia Y., Wu K. (2017). An investigation of the health effects caused by exposure to arsenic from drinking water and coal combustion: Arsenic exposure and metabolism. Environ. Sci. Pollut. Res..

[76] Rasheed H., Kay P., Slack R., Gong Y.Y., Carter A. (2017). Human exposure assessment of different arsenic species in household water sources in a high risk arsenic area. Sci. Total. Environ..

[77] Chang J.Y., Ahn S.C., Lee J.S., Kim J.-Y., Jung A.-R., Park J., Choi J.-W., Yu S.D. (2019). Exposure assessment for the abandoned metal mine area contaminated by arsenic. Environ. Geochem. Health.

[78] Signes-Pastor A.J., Vioque J., Navarrete-Muñoz E.M., Carey M., García-Villarino M., Fernández-Somoano A., Tardón A., Santa-Marina L., Irizar A., Casas M. (2019). Inorganic arsenic exposure and neuropsychological development of children of 4–5 years of age living in Spain. Environ. Res..

[79] Samal A.C., Kar S., Bhattacharya P., Santra S. (2011). Human exposure to arsenic through foodstuffs cultivated using arsenic contaminated groundwater in areas of West Bengal, India. J. Environ. Sci. Health Part A Tox Hazard. Subst. Environ. Eng..

[80] Menon M., Sarkar B., Hufton J., Reynolds C., Reina S.V., Young S. (2020). Do arsenic levels in rice pose a health risk to the UK population?. Ecotoxicol. Environ. Saf..

[81] Liao N., Seto E., Eskenazi B., Wang M., Li Y., Hua J. (2018). A Comprehensive Review of Arsenic Exposure and Risk from Rice and a Risk Assessment among a Cohort of Adolescents in Kunming, China. Int. J. Environ. Res. Public Health.

[82] Oguri T., Yoshinaga J. (2014). Daily Inorganic Arsenic Intake of the Japanese Estimated by a Probabilistic Approach. Nippon Eiseigaku Zasshi Jpn. J. Hyg..

[83] Rasheed H., Kay P., Slack R., Gong Y.Y. (2018). Arsenic species in wheat, raw and cooked rice: Exposure and associated health implications. Sci. Total. Environ..

[84] Xue L., Zhao Z., Zhang Y., Liao J., Wu M., Wang M., Sun J., Gong H., Guo M., Li S. (2020). Dietary exposure to arsenic and human health risks in western Tibet. Sci. Total Environ..

[85] Raeeszadeh M., Gravandi H., Akbari A. (2022). Determination of some heavy metals levels in the meat of animal species (sheep, beef, turkey, and ostrich) and carcinogenic health risk assessment in Kurdistan province in the west of Iran. Environ. Sci. Pollut. Res..

[86] Vahter M. (2002). Mechanisms of arsenic biotransformation. Toxicology.

[87] National Research Council (US) Subcommittee on Arsenic in Drinking Water (1999). Disposition of Inorganic Arsenic.

[88] Lindgren A., Vahter M., Dencker L. (1982). Autoradiographic Studies on the Distribution of Arsenic in Mice and Hamsters Administered 74As-Arsenite or -Arsenate. Acta Pharmacol. Toxicol..

[89] Lindgren A., Danielsson B.R.G., Dencker L., Vahter M. (1984). Embryotoxicity of Arsenite and Arsenate: Distribution in Pregnant Mice and Monkeys and Effects on Embryonic Cells in Vitro. Acta Pharmacol. Toxicol..

[90] Vahter M., Marafante E., Lindgren A., Dencker L. (1982). Tissue distribution and subcellular binding of arsenic in Marmoset monkeys after injection of 74As-Arsenite. Arch. Toxicol..

[91] Garbinski L.D., Rosen B.P., Chen J. (2019). Pathways of arsenic uptake and efflux. Environ. Int..

[92] Roggenbeck B.A., Banerjee M., Leslie E.M. (2016). Cellular arsenic transport pathways in mammals. J. Environ. Sci..

[93] Hughes M.F. (2002). Arsenic toxicity and potential mechanisms of action. Toxicol. Lett..

[94] Rehman K., Naranmandura H. (2012). Arsenic metabolism and thioarsenicals. Metallomics.

[95] Suzuki K., Ogra Y. (2004). Distributions and chemical forms of arsenic after intravenous administration of dimethylarsinic and monomethylarsonic acids to rats. Toxicol. Appl. Pharmacol..

[96] Kligerman A.D., Doerr C.L., Tennant A.H., Harrington-Brock K., Allen J.W., Winkfield E., Poorman-Allen P., Kundu B., Funasaka K., Roop B.C. (2003). Methylated trivalent arsenicals as candidate ultimate genotoxic forms of arsenic: Induction of chromosomal mutations but not gene mutations. Environ. Mol. Mutagen..

[97] Mass M.J., Tennant A., Roop B.C., Cullen W.R., Styblo M., Thomas D.J., Kligerman A.D. (2001). Methylated Trivalent Arsenic Species Are Genotoxic. Chem. Res. Toxicol..

[98] Braman R.S., Foreback C.C. (1973). Methylated Forms of Arsenic in the Environment. Science.

[99] Chouchane S., Snow E.T. (2001). In Vitro Effect of Arsenical Compounds on Glutathione-Related Enzymes. Chem. Res. Toxicol..

[100] Yamanaka K., Hayashi H., Tachikawa M., Kato K., Hasegawa A., Oku N., Okada S. (1997). Metabolic methylation is a possible genotoxicity-enhancing process of inorganic arsenics. Mutat. Res. Toxicol. Environ. Mutagen..

[101] Ahmad S., Kitchin K.T., Cullen W.R. (2000). Arsenic Species That Cause Release of Iron from Ferritin and Generation of Activated Oxygen. Arch. Biochem. Biophys..

[102] Corsini E., Asti L., Viviani B., Marinovich M., Galli C.L. (1999). Sodium Arsenate Induces Overproduction of Interleukin-1α in Murine Keratinocytes: Role of Mitochondria. J. Investig. Dermatol..

[103] Tsai C.-H., Yang M.-H., Hung A.C., Wu S.-C., Chiu W.-C., Hou M.-F., Tyan Y.-C., Wang Y.-M., Yuan S.-S.F. (2015). Identification of Id1 as a downstream effector for arsenic-promoted angiogenesis via PI3K/Akt, NF-κB and NOS signaling. Toxicol. Res..

[104] Huang Q., Luo L., Alamdar A., Zhang J., Liu L., Tian M., Eqani S.A.M.A.S., Shen H. (2016). Integrated proteomics and metabolomics analysis of rat testis: Mechanism of arsenic-induced male reproductive toxicity. Sci. Rep..

[105] Huang Y.-C., Yu H.-S., Chai C.-Y. (2015). Proteins in the ERK pathway are affected by arsenic-treated cells. Toxicol. Res..

[106] Chen J., Fok K.L., Chen H., Zhang X.H., Xu W.M., Chan H.C. (2012). Cryptorchidism-induced CFTR down-regulation results in disruption of testicular tight junctions through up-regulation of NF- B/COX-2/PGE2. Hum. Reprod..

[107] Xia Z.-P., Zheng X.-M., Zheng H., Liu X.-J., Liu G.-Y., Wang X. (2012). Downregulation of cold-inducible RNA-binding protein activates mitogen-activated protein kinases and impairs spermatogenic function in mouse testes. Asian J. Androl..

[108] Danielsson B.R.G., Dencker L., Lindgren A., Tjälve H. (1984). Accumulation of Toxic Metals in Male Reproduction Organs. Arch. Toxicol. Suppl..

[109] Pant N., Kumar R., Murthy R.C., Srivastava S.P. (2001). Male reproductive effect of arsenic in mice. BioMetals.

[110] Zubair M., Ahmad M., Qureshi Z.I. (2017). Review on Arsenic-Induced Toxicity in Male Reproductive System and Its Amelioration. Andrologia.

[111] Kumar A., Raj V., Srivastava A., Ali M., Ghosh A.K., Rachamalla M., Kumar D. (2022). Autophagy in arsenic exposed population and cancer patients. Autophagy and Metabolism.

[112] Roy S., Bhattacharya S. (2006). Arsenic-induced histopathology and synthesis of stress proteins in liver and kidney of Channa punctatus. Ecotoxicol. Environ. Saf..

[113] Ahmed K., Mamun H.A., Parvin E., Akter M.S., Khan M.S. (2013). Arsenic induced toxicity and histopathological changes in gill and liver tissue of freshwater fish, tilapia (Oreochromis mossambicus). Exp. Toxicol. Pathol..

[114] Manthari R.K., Tikka C., Ommati M.M., Niu R., Sun Z., Wang J., Zhang J., Wang J. (2018). Arsenic-Induced Autophagy in the Developing Mouse Cerebellum: Involvement of the Blood-Brain Barrier’s Tight-Junction Proteins and the PI3K-Akt-MTOR Signaling Pathway. J. Agric. Food Chem..

[115] Manthari R.K., Tikka C., Ommati M.M., Niu R., Sun Z., Wang J., Zhang J., Wang J. (2018). Arsenic Induces Autophagy in De-velopmental Mouse Cerebral Cortex and Hippocampus by Inhibiting PI3K/Akt/MTOR Signaling Pathway: Involvement of Blood–Brain Barrier’s Tight Junction Proteins. Arch. Toxicol..

[116] Ommati M.M., Heidari R., Zamiri M.J., Sabouri S., Zaker L., Farshad O., Jamshidzadeh A., Mousapour S. (2020). The Footprints of Oxidative Stress and Mitochondrial Impairment in Arsenic Trioxide-Induced Testosterone Release Suppression in Pubertal and Mature F1-Male Balb/c Mice via the Downregulation of 3β-HSD, 17β-HSD, and CYP11a Expression. Biol. Trace Elem. Res..

[117] Acevedo-Rodriguez A., Kauffman A.S., Cherrington B.D., Borges C.S., Roepke T.A., Laconi M. (2018). Emerging Insights into Hypothalamic-Pituitary-Gonadal (HPG) Axis Regulation and Interaction with Stress Signaling. J. Neuroendocrinol..

[118] Chiaverini N., de Ley M. (2010). Protective Effect of Metallothionein on Oxidative Stress-Induced DNA Damage. Free Radic. Res..

[119] Siu E., Mruk D.D., Porto C.S., Cheng C.Y. (2009). Cadmium-induced testicular injury. Toxicol. Appl. Pharmacol..

[120] Dalton T., Fu K., Enders G.C., Palmiter R.D., Andrews G.K. (1996). Analysis of the effects of overexpression of metallothionein-I in transgenic mice on the reproductive toxicology of cadmium. Environ. Health Perspect..

[121] Cheng C.Y., Mruk D.D. (2011). The Blood-Testis Barrier and Its Implications for Male Contraception. Pharmacol. Rev..

[122] Sanghamitra S., Hazra J., Upadhyay S.N., Singh R.K., Amal R.C. (2008). Arsenic induced toxicity on testicular tissue of mice. Indian J. Physiol. Pharmacol..

[123] Wang L., Hao J., Hu J., Pu J., Lü Z., Zhao L., Wang Q., Yu Q., Wang Y., Li G. (2012). Protective Effects of Ginsenosides against Bisphenol A-Induced Cytotoxicity in 15P-1 Sertoli Cells via Extracellular Signal-Regulated Kinase 1/2 Signalling and Antioxidant Mechanisms. Basic Clin. Pharmacol. Toxicol..

[124] Momeni H.R., Oryan S., Eskandari N. (2012). Effect of vitamin E on sperm number and testis histopathology of sodium arsenite-treated rats. Reprod. Biol..

[125] de Medeiros P., Samelo R.R., Silva A.P.G., Santiago M., Duarte F., Castro ., Perobelli J.E. (2018). Prepubertal exposure to low doses of sodium arsenite impairs spermatogenesis and epididymal histophysiology in rats. Environ. Toxicol..

[126] Anwar N., Qureshi I.Z., Spears N., Lopes F. (2020). In vitro administration of sodium arsenite in mouse prepubertal testis induces germ cell loss and apoptosis. Toxicol. Vitro.

[127] Souza A.C.F., Marchesi S.C., Lima G.D.D.A., Ferraz R.P., Santos F.C., da Matta S.L.P., Machado-Neves M. (2015). Effects of Sodium Arsenite and Arsenate in Testicular Histomorphometry and Antioxidants Enzymes Activities in Rats. Biol. Trace Element Res..

[128] Chiou T.-J., Chu S.-T., Tzeng W.-F., Huang Y.-C., Liao C.-J. (2008). Arsenic Trioxide Impairs Spermatogenesis via Reducing Gene Expression Levels in Testosterone Synthesis Pathway. Chem. Res. Toxicol..

[129] Wang Y.-X., Wang P., Feng W., Liu C., Yang P., Chen Y.-J., Sun L., Sun Y., Yue J., Gu L.-J. (2017). Relationships between seminal plasma metals/metalloids and semen quality, sperm apoptosis and DNA integrity. Environ. Pollut..

[130] Lima G.D.D.A., Sertorio M.N., Souza A.C.F., Menezes T.P., Mouro V.G.S., Gonçalves N.M., de Oliveira J.M., Henry M., Machado-Neves M. (2018). Fertility in male rats: Disentangling adverse effects of arsenic compounds. Reprod. Toxicol..

[131] da Silva R.F., Borges C.D.S., Lamas C.D.A., Cagnon V.H.A., Kempinas W.D.G. (2017). Arsenic trioxide exposure impairs testicular morphology in adult male mice and consequent fetus viability. J. Toxicol. Environ. Health Part A Curr. Issues.

[132] Han Y., Liang C., Yu Y., Manthari R.K., Cheng C., Tan Y., Li X., Tian X., Fu W., Yang J. (2020). Chronic arsenic exposure lowered sperm motility via impairing ultra-microstructure and key proteins expressions of sperm acrosome and flagellum formation during spermiogenesis in male mice. Sci. Total Environ..

[133] Han Y., Liang C., Manthari R.K., Yu Y., Gao Y., Liu Y., Jiang S., Tikka C., Wang J., Zhang J. (2019). Arsenic influences spermatogenesis by disorganizing the elongation of spermatids in adult male mice. Chemosphere.

[134] Kesari V.P., Kumar A., Khan P.K. (2014). Induction of sperm impairments in mice as a sensitive biomarker of arsenic toxicity. Environ. Monit. Assess..

[135] Souza A.C.F., Bastos D.S.S., Sertorio M.N., Santos F.C., Ervilha L.O.G., de Oliveira L.L., Machado-Neves M. (2019). Combined effects of arsenic exposure and diabetes on male reproductive functions. Andrology.

[136] Guvvala P.R., Sellappan S., Parameswaraiah R.J. (2016). Impact of arsenic(V) on testicular oxidative stress and sperm functional attributes in Swiss albino mice. Environ. Sci. Pollut. Res..

[137] Rodriguez K.F., Mellouk N., Ungewitter E.K., Nicol B., Liu C., Brown P.R., Willson C.J., Yao H.H.-C. (2020). In utero exposure to arsenite contributes to metabolic and reproductive dysfunction in male offspring of CD-1 mice. Reprod. Toxicol..

[138] Nava-Rivera L.E., Betancourt-Martínez N.D., Lozoya-Martínez R., Carranza-Rosales P., Guzmán-Delgado N.E., Carranza-Torres I.E., Delgado-Aguirre H., Zambrano-Ortíz J.O., Morán-Martínez J. (2021). Transgenerational effects in DNA methylation, genotoxicity and reproductive phenotype by chronic arsenic exposure. Sci. Rep..

[139] Souza A.C.F., Machado-Neves M., Bastos D.S.S., Santos F.C., Ervilha L.O.G., Coimbra J.L.D.P., Araújo L.D.S., de Oliveira L.L., Guimarães S.E.F. (2020). Impact of prenatal arsenic exposure on the testes and epididymides of prepubertal rats. Chem. Interact..

[140] Couto-Santos F., Viana A.G.D.A., Souza A.C.F., Dutra A.A.D.A., Mendes T.A.D.O., Ferreira A.T.D.S., Aguilar J.E.P., Oliveira L.L., Machado-Neves M. (2021). Prepubertal arsenic exposure alters phosphoproteins profile, quality, and fertility of epididymal spermatozoa in sexually mature rats. Toxicology.

[141] Souza A.C.F., Marchesi S.C., Ferraz R.P., Lima G.D.D.A., de Oliveira J.A., Machado-Neves M. (2016). Effects of sodium arsenate and arsenite on male reproductive functions in Wistar rats. J. Toxicol. Environ. Health Part A.

[142] Ram A.K.S.S., Reddy K.P., Girish B.P., Supriya C., Reddy P.S. (2018). Arsenic aggravated reproductive toxicity in male rats exposed to lead during the perinatal period. Toxicol. Res..

[143] Liu P., Li R., Tian X., Zhao Y., Li M., Wang M., Ying X., Yuan J., Xie J., Yan X. (2021). Co-exposure to fluoride and arsenic disrupts intestinal flora balance and induces testicular autophagy in offspring rats. Ecotoxicol. Environ. Saf..

[144] Yilmaz B.O., Yildizbayrak N., Erkan M. (2018). Sodium arsenite-induced detriment of cell function in Leydig and Sertoli cells: The potential relation of oxidative damage and antioxidant defense system. Drug Chem. Toxicol..

[145] Zeng Q., Yi H., Huang L., An Q., Wang H. (2018). Reduced testosterone and Ddx3y expression caused by long-term exposure to arsenic and its effect on spermatogenesis in mice. Environ. Toxicol. Pharmacol..

[146] Wu S., Zhong G., Wan F., Jiang X., Tang Z., Hu T., Rao G., Lan J., Hussain R., Tang L. (2021). Evaluation of toxic effects induced by arsenic trioxide or/and antimony on autophagy and apoptosis in testis of adult mice. Environ. Sci. Pollut. Res..

[147] Alamdar A., Tian M., Huang Q., Du X., Zhang J., Liu L., Shah S.T.A., Shen H. (2019). Enhanced histone H3K9 tri-methylation suppresses steroidogenesis in rat testis chronically exposed to arsenic. Ecotoxicol. Environ. Saf..

[148] Couto-Santos F., Souza A.C.F., Bastos D.S.S., Ervilha L.O.G., Dias F.C.R., Araújo L.D.S., Guimarães S.E.F., de Oliveira L.L., Machado-Neves M. (2020). Prepubertal exposure to arsenic alters male reproductive parameters in pubertal and adult rats. Toxicol. Appl. Pharmacol..

[149] Sun X., Li S., He Y., Zhao H., Wang Y., Zeng X., Xing M. (2017). Arsenic-induced testicular toxicity in Gallus gallus: Expressions of inflammatory cytokines and heat shock proteins. Poult. Sci..

[150] Shao Y., Zhao H., Wang Y., Liu J., Li J., Chai H., Xing M. (2017). Arsenic and/or copper caused inflammatory response via activation of inducible nitric oxide synthase pathway and triggered heat shock protein responses in testis tissues of chicken. Environ. Sci. Pollut. Res..

[151] Shao Y., Zhao H., Wang Y., Liu J., Li J., Luo L., Xing M. (2018). The apoptosis in arsenic-induced oxidative stress is associated with autophagy in the testis tissues of chicken. Poult. Sci..

[152] Liang C., Feng Z., Manthari R.K., Wang C., Han Y., Fu W., Wang J., Zhang J. (2020). Arsenic induces dysfunctional autophagy via dual regulation of mTOR pathway and Beclin1-Vps34/PI3K complex in MLTC-1 cells. J. Hazard. Mater..

[153] Alamdar A., Xi G., Huang Q., Tian M., Eqani S.A.M.A.S., Shen H. (2017). Arsenic activates the expression of 3β-HSD in mouse Leydig cells through repression of histone H3K9 methylation. Toxicol. Appl. Pharmacol..

[154] Chen H., Liu G., Qiao N., Kang Z., Hu L., Liao J., Yang F., Pang C., Liu B., Zeng Q. (2019). Toxic effects of arsenic trioxide on spermatogonia are associated with oxidative stress, mitochondrial dysfunction, autophagy and metabolomic alterations. Ecotoxicol. Environ. Saf..

[155] Jomova K., Jenisova Z., Feszterova M., Baros S., Liska J., Hudecova D., Rhodes C.J., Valko M. (2011). Arsenic: Toxicity, Oxidative Stress and Human Disease. J. Appl. Toxicol..

[156] Tariba Lovaković B. (2020). Cadmium, Arsenic, and Lead: Elements Affecting Male Reproductive Health. Curr. Opin. Toxicol..

[157] Anwar N., Qureshi I.Z. (2019). In vitro application of sodium arsenite to mice testicular and epididymal organ cultures induces oxidative, biochemical, hormonal, and genotoxic stress. Toxicol. Ind. Health.

[158] Ji P.-Y., Li Z.-Y., Wang H., Dong J.-T., Li X.-J., Yi H.-L. (2019). Arsenic and sulfur dioxide co-exposure induce renal injury via activation of the NF-κB and caspase signaling pathway. Chemosphere.

[159] Zhong G., Wan F., Wu S., Jiang X., Tang Z., Zhang X., Huang R., Hu L. (2021). Arsenic or/and antimony induced mitophagy and apoptosis associated with metabolic abnormalities and oxidative stress in the liver of mice. Sci. Total Environ..

[160] Qi Y., Li H., Zhang M., Zhang T., Frank J., Chen G. (2014). Autophagy in Arsenic Carcinogenesis. Exp. Toxicol. Pathol..

[161] Zeinvand-Lorestani M., Kalantari H., Khodayar M.J., Teimoori A., Saki N., Ahangarpour A., Rahim F., Alboghobeish S. (2018). Autophagy upregulation as a possible mechanism of arsenic induced diabetes. Sci. Rep..

[162] Tang Q., Bai L., Zou Z., Meng P., Xia Y., Cheng S., Mu S., Zhou J., Wang X., Qin X. (2018). Ferroptosis is newly characterized form of neuronal cell death in response to arsenite exposure. NeuroToxicology.

[163] Meng P., Zhang S., Jiang X., Cheng S., Zhang J., Cao X., Qin X., Zou Z., Chen C. (2020). Arsenite induces testicular oxidative stress in vivo and in vitro leading to ferroptosis. Ecotoxicol. Environ. Saf..

[164] Wang H., Li J., Zhang X., Zhu P., Hao J.-H., Tao F.-B., Xu D.-X. (2018). Maternal serum arsenic level during pregnancy is positively associated with adverse pregnant outcomes in a Chinese population. Toxicol. Appl. Pharmacol..

[165] Li Y.Y., Chen S.W., Zhao F., Zhang H.M., Zhang W.L., Qu Y.L., Liu Y.C., Gu H., Cai J.Y., Cao Z.J. (2019). Association of arsenic with unexplained recurrent spontaneous abortion: A case-control study. Zhonghua Yu Fang Yi Xue Za Zhi.

[166] Liang C., Han Y., Ma L., Wu X., Huang K., Yan S., Li Z., Xia X., Pan W., Sheng J. (2020). Low levels of arsenic exposure during pregnancy and maternal and neonatal thyroid hormone parameters: The determinants for these associations. Environ. Int..

[167] Zubair M., Ahmad M., Saleemi M.K., Gul S.T., Ahmad N., Umar S. (2016). Protective effects of vitamin E on sodium arsenite-induced toxicity, testicular measurements and histopathological studies of testes in Teddy goat bucks. Andrologia.

[168] Zhong Q., Cui Y., Wu H., Niu Q., Lu X., Wang L., Huang F. (2019). Association of maternal arsenic exposure with birth size: A systematic review and meta-analysis. Environ. Toxicol. Pharmacol..

[169] Almberg K.S., Turyk M.E., Jones R.M., Rankin K., Freels S., Graber J.M., Stayner L.T. (2017). Arsenic in drinking water and adverse birth outcomes in Ohio. Environ. Res..

[170] Yin G., Xia L., Hou Y., Li Y., Cao D., Liu Y., Chen J., Liu J., Zhang L., Yang Q. (2021). Transgenerational male reproductive effect of prenatal arsenic exposure: Abnormal spermatogenesis with Igf2/H19 epigenetic alteration in CD1 mouse. Int. J. Environ. Health Res..

[171] Tian M., Wang Y.-X., Wang X., Wang H., Liu L., Zhang J., Nan B., Shen H., Huang Q. (2020). Environmental doses of arsenic exposure are associated with increased reproductive-age male urinary hormone excretion and in vitro Leydig cell steroidogenesis. J. Hazard. Mater..

[172] Shen H., Xu W., Zhang J., Chen M., Martin F.L., Xia Y., Liu L., Dong S., Zhu Y.-G. (2013). Urinary Metabolic Biomarkers Link Oxidative Stress Indicators Associated with General Arsenic Exposure to Male Infertility In a Han Chinese Population. Environ. Sci. Technol..

[173] He Y., Zou L., Luo W., Yi Z., Yang P., Yu S., Liu N., Ji J., Guo Y., Liu P. (2019). Heavy metal exposure, oxidative stress and semen quality: Exploring associations and mediation effects in reproductive-aged men. Chemosphere.

[174] Sharma G., Kumar M. (2012). Antioxidant and modulatory role of Chlorophytum borivilianum against arsenic induced testicular impairment. J. Environ. Sci..

[175] Prathima P., Pavani R., Sukeerthi S., Sainath S.B. (2017). α-Lipoic acid inhibits testicular and epididymal oxidative damage and improves fertility efficacy in arsenic-intoxicated rats. J. Biochem. Mol. Toxicol..

[176] Biswas S., Mukhopadhyay P.K. (2020). Casein- and pea-enriched high-protein diet can take care of the reprotoxic effects of arsenic in male rats. Andrologia.

[177] Samadder A., Das J., Das S., Khuda-Bukhsh A.R. (2012). Dihydroxy-isosteviol-methyl-ester, an active biological component of Pulsatilla nigricans, reduces arsenic induced cellular dysfunction in testis of male mice. Environ. Toxicol. Pharmacol..

[178] Mehrzadi S., Bahrami N., Mehrabani M., Motevalian M., Mansouri E., Goudarzi M. (2018). Ellagic acid: A promising protective remedy against testicular toxicity induced by arsenic. Biomed. Pharmacother..

[179] Guvvala P.R., Ravindra J.P., Selvaraju S., Arangasamy A., Venkata K.M. (2018). Ellagic and ferulic acids protect arsenic-induced male reproductive toxicity via regulating Nfe2l2, Ppargc1a and StAR expressions in testis. Toxicology.

[180] Li S.G., Xu S.Z., Niu Q., Ding Y.S., Pang L.J., Ma R.L., Jing M.X., Wang K., Ma X.M., Feng G.L. (2015). Lutein alleviates arsenic-induced reproductive toxicity in male mice via Nrf2 signaling. Hum. Exp. Toxicol..

[181] Li S.G., Ding Y.S., Niu Q., Xu S.Z., Pang L.J., Ma R.L., Jing M.X., Feng G.L., Liu J.M., Guo S.X. (2015). Grape Seed Proanthocyanidin Extract Alleviates Arsenic-induced Oxidative Reproductive Toxicity in Male Mice. Biomed. Environ. Sci..

[182] Ince S., Avdatek F., Demirel H.H., Arslan-Acaroz D., Goksel E., Kucukkurt I. (2015). Ameliorative effect of polydatin on oxidative stress-mediated testicular damage by chronic arsenic exposure in rats. Andrologia.

[183] Guvvala P.R., Ravindra J.P., Rajani C.V., Sivaram M., Selvaraju S. (2017). Protective role of epigallocatechin-3-gallate on arsenic induced testicular toxicity in Swiss albino mice. Biomed. Pharmacother..

[184] Reddy P.S., Rani G.P., Sainath S., Meena R., Supriya C. (2011). Protective effects of N-acetylcysteine against arsenic-induced oxidative stress and reprotoxicity in male mice. J. Trace Elements Med. Biol..

[185] El-Khadragy M.F., Al-Megrin W.A., Alomar S., Alkhuriji A.F., Metwally D.M., Mahgoub S., Amin H.K., Habotta O.A., Moneim A.E.A., Albeltagy R.S. (2020). Chlorogenic acid abates male reproductive dysfunction in arsenic-exposed mice via attenuation of testicular oxido-inflammatory stress and apoptotic responses. Chem. Interact..

[186] Ola-Davies O., Ajani O.S. (2016). Semen characteristics and sperm morphology of Pistia stratiotes Linn. (Araceae) protected male albino rats (Wistar strain) exposed to sodium arsenite. J. Complement. Integr. Med..

[187] Adedara I.A., Adebowale A.A., Atanda O.E., Fabunmi A.T., Ayenitaju A.C., Rocha J.B., Farombi E.O. (2019). Selenium abates reproductive dysfunction via attenuation of biometal accumulation, oxido-inflammatory stress and caspase-3 activation in male rats exposed to arsenic. Environ. Pollut..

[188] da Silva R.F., Borges C.D.S., e Silva P.V., Missassi G., Kiguti L.R.A., Pupo A.S., Junior F.B., Anselmo-Franci J.A., Kempinas W.D.G. (2015). The Coadministration of N-Acetylcysteine Ameliorates the Effects of Arsenic Trioxide on the Male Mouse Genital System. Oxidat. Med. Cell. Longev..

[189] Uygur R., Aktas C., Caglar V., Uygur E., Erdogan H., Ozen O.A. (2013). Protective effects of melatonin against arsenic-induced apoptosis and oxidative stress in rat testes. Toxicol. Ind. Health.

[190] Fouad A.A., Al-Sultan A.I., Yacoubi M.T. (2011). Coenzyme Q10 counteracts testicular injury induced by sodium arsenite in rats. Eur. J. Pharmacol..

[191] Baltaci B.B., Uygur R., Caglar V., Aktas C., Aydin M., Ozen O.A. (2016). Protective effects of quercetin against arsenic-induced testicular damage in rats. Andrologia.

[192] Jahan S., Rehman S., Ullah H., Munawar A., Ain Q.U., Iqbal T. (2015). Ameliorative effect of quercetin against arsenic-induced sperm DNA damage and daily sperm production in adult male rats. Drug Chem. Toxicol..

[193] Jahan S., Iftikhar N., Ullah H., Rukh G., Hussain I. (2014). Alleviative effect of quercetin on rat testis against arsenic: A histological and biochemical study. Syst. Biol. Reprod. Med..

[194] Durg S., Shivaram S.B., Bavage S. (2018). Withania somnifera (Indian ginseng) in male infertility: An evidence-based systematic review and meta-analysis. Phytomedicine.

[195] Altoé L.S., Reis I.B., Gomes M., Dolder H., Pirovani J.M. (2016). Could vitamin C and zinc chloride protect the germ cells against sodium arsenite?. Hum. Exp. Toxicol..

[196] Ajibade T.O., Olayemi F.O. (2020). Polyphenol-rich fraction ofAlchornea cordifolialeaf ameliorates arsenite-induced infertility in male rats. Andrologia.

[197] Ogunlade B., Adelakun S.A., Ukwenya V., Elemoso T. (2021). Potentiating response of D- Ribose-L-Cysteine on Sodium arsenate- induced hormonal imbalance, spermatogenesis impairments and histomorphometric alterations in adult male Wistar rat. JBRA Assist. Reprod..

[198] Sampayo-Reyes A., Taméz-Guerra R.S., de León M.B., Vargas-Villarreal J., Lozano-Garza H.G., Rodríguez-Padilla C., Cortés C., Marcos R., Hernández A. (2017). Tocopherol and selenite modulate the transplacental effects induced by sodium arsenite in hamsters. Reprod. Toxicol..

[199] Mwaeni V.K., Nyariki J.N., Jillani N., Omwenga G., Ngugi M., Isaac A.O. (2021). Coenzyme Q10 Protected against Arsenite and Enhanced the Capacity of 2,3-Dimercaptosuccinic Acid to Ameliorate Arsenite-Induced Toxicity in Mice. BMC Pharmacol. Toxicol..

[200] Zubair M., Ahmad M., Jamil H., Deeba F. (2016). Toxic effects of arsenic on semen and hormonal profile and their amelioration with vitamin E in Teddy goat bucks. Andrologia.

[201] Raeeszadeh M., Karimfar B., Amiri A.A., Akbari A. (2021). Protective Effect of Nano-Vitamin C on Infertility due to Oxidative Stress Induced by Lead and Arsenic in Male Rats. J. Chem..

[202] Raeeszadeh M., Karimi P., Khademi N., Mortazavi P. (2022). The Effect of Broccoli Extract in Arsenic-Induced Experimental Poisoning on the Hematological, Biochemical, and Electrophoretic Parameters of the Liver and Kidney of Rats. Evid.-Based Complement. Altern. Med..

